# Constructing a 10‐core genes panel for diagnosis of pediatric sepsis

**DOI:** 10.1002/jcla.23680

**Published:** 2020-12-03

**Authors:** Xiaojie Zhou, Yumin Wang, Jie Chen, Jingye Pan

**Affiliations:** ^1^ Department of Intensive Care Unit The First Affiliated Hospital of Wenzhou Medical University Wenzhou China; ^2^ Department of Laboratory Medicine The First Affiliated Hospital of Wenzhou Medical University Wenzhou China

**Keywords:** core gene, diagnosis, panel, pediatric sepsis

## Abstract

**Background:**

The lack of sensitivity and specificity of most biomarkers or the lack of relevant studies to demonstrate their effectiveness in sepsis.

**Methods:**

Downloaded three sets of sepsis expression data (GSE13904, GSE25504, GSE26440) from GEO. Then, using the R limma package and WGCNA analysis tocore genes. Finally, the value of these core genes was confirmed by clinical samples.

**Results:**

Compared to normal samples, we obtain many abnormally expressed genes in the pediatric sepsis. WGCNA co‐expression analysis showed that genes from blue and turquoise module were close correlation with pediatric sepsis. The top 20 genes (TIMP2, FLOT1, HCK, NCF4, SERPINA1, IL17RA, PGD, PRKCD, GLT1D1, ALOX5, SIRPA, DOK3, ITGAM, S100A11, ZNF438, PLIN3, LTB4R, TSPO, MAPK14, GAS7) of the blue module of pediatric sepsis were mainly enriched in neutrophil degranulation, etc The top 20 genes (TBC1D4, NOL11, NLRC3, ZNF121, DYRK2, ABCE1, MAGEH1, TMEM263, MCUB, MALT1, DDHD2, TRAC, NOC3L, LCK, TRMT61B, ZNF260, ENOPH1, LOC93622, NAE1, TRBC1) for turquoise module were mainly enriched in rRNA‐containing ribonucleoprotein complexes exported from the nucleus, etc The selected hub gene of pediatric sepsis was combined with the markers of cell surface and found 10 core genes (HCK, PRKCD, SIRPA, DOK3, ITGAM, LTB4R, MAPK14, MALT1, NLRC3, LCK). ROC showed that AUC of the 10 core genes for diagnosis of pediatric sepsis was above 0.9.

**Conclusion:**

There were many abnormally expressed genes in patients with pediatric sepsis. The panel constructed by the 10 core genes was expected to become a biomarker panel for clinical application of pediatric sepsis.

## Graphical abstract

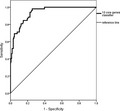

## 1 INTRODUCTION

The initial definition of sepsis (Sepsis 1.0),^1^ the systemic inflammatory response syndrome (SIRS) caused by infection. According to the new definition of sepsis 3.0, sepsis was the host's dysfunctional response to infection leading to life‐threatening organ dysfunction.^2^ In the age of intensive care management, sepsis remains one of the leading causes of death in intensive care units (ICUs).^3^ The complex pathophysiological mechanism of sepsis leads to the release of many biomarkers. By measuring more biomarkers, the host's response to infection can be better evaluated to better guide clinical treatment.^4^

In the past two decades, many sepsis‐related markers have been reported, and these biomarkers have been diagnosed due to a lack of consistent baseline in the study. The accuracy of these biomarkers was still unclear.^5^ Many scholars were dedicated to the study of different types of biomarkers for early diagnosis of pediatric sepsis. And assess the role of prognosis. In general, biomarkers of pediatric sepsis can be classified according to their role as systemic inflammatory mediators^6^: ① molecules expressed on the phagocytic membrane [myeloid cell trigger receptor‐1 (TREM‐1), CD14 receptor body, CD163 receptor]. ② cytokines, such as interleukin (IL)‐6, IL‐8, IL‐10. ③ acute phase proteins, such as procalcitonin (PCT) or C‐reactive protein (CRP). However, the lack of sensitivity and specificity of most biomarkers or the lack of relevant studies demonstrates their effectiveness. Therefore, it was particularly urgent to look for more specific and more sensitive markers for predicting pediatric sepsis. We expect to obtain some genes as pediatric sepsis markers of diagnosis and prognosis.

## 2 MATERIALS AND METHODS

### 2.1 Study population

Three sets of pediatric sepsis gene expression data and their corresponding platform annotation data were downloaded from the GEO database: GSE13904 (GPL570 platform), including 209 pediatric sepsis samples and 18 normal samples; GSE25504 (GPL570, GPL6947, GPL13667 platform), including 44 pediatric sepsis samples, 44 normal samples; GSE26440 (GPL570 platform), including 98 pediatric sepsis samples and 32 normal samples.

### 2.2 Bioinformation analysis

**Figure 1:**
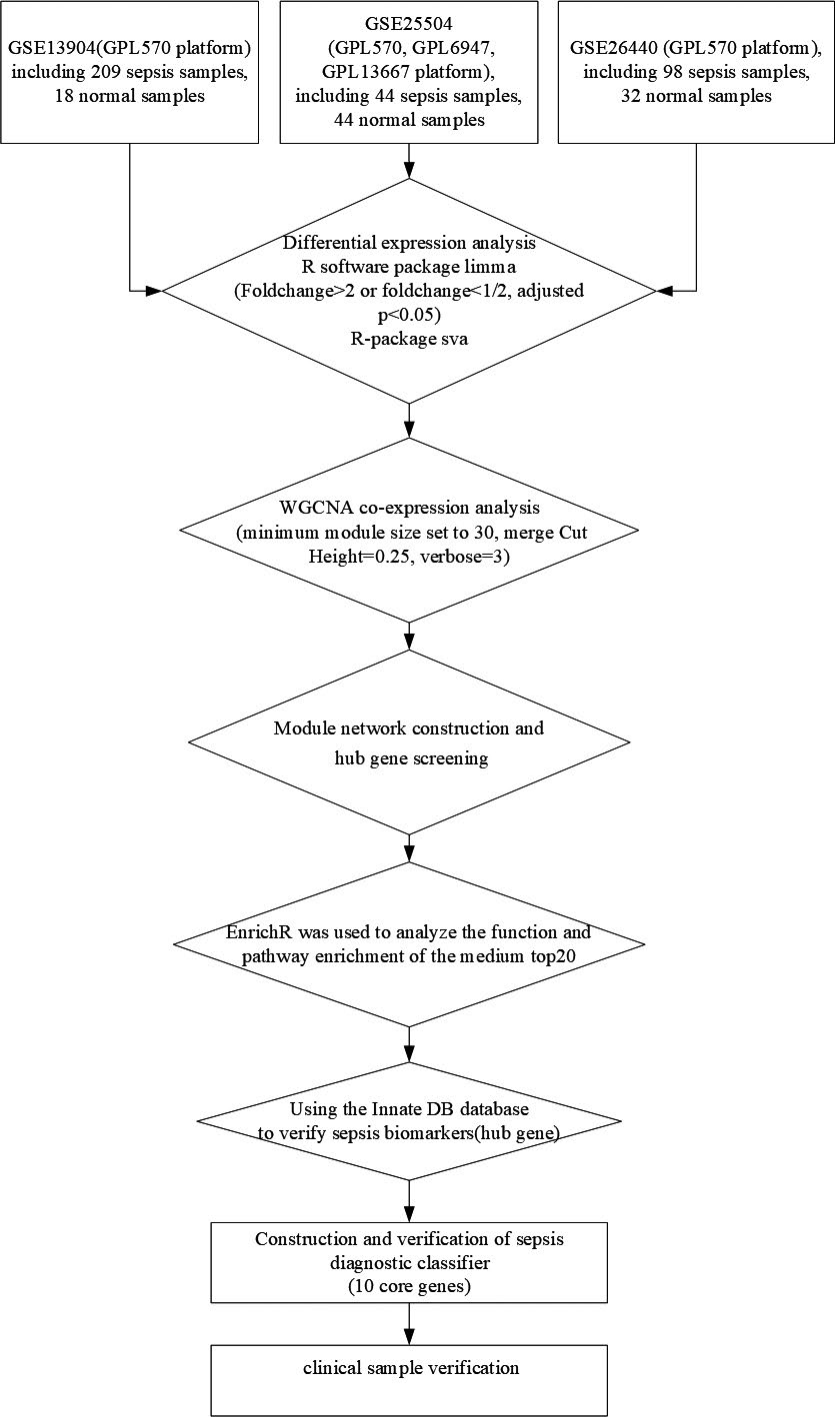
Flow chart of the study

The work flow chart was shown in Figure 1.

### 2.3 Clinical patient verification

To analyze the clinical data of 48 patients with pediatric sepsis who were admitted to the Department of Newborn Critical Care Medicine (NICU) of the First Affiliated Hospital of Wenzhou Medical University from July 2010 to January 2019. In the study group, there were 48 cases, 23 males and 25 females; of which 15 were severely traumatized, 10 were abdominal injuries, 5 had larger burns, 10 were severe pneumonia, and 8 were acute peritonitis; aged 2–13 years old, mean age (5.8 ± 1.3) years old; the length of hospitalization was 10–28 days; the average length of hospitalization was (16.6 ± 5.2) days. In the control group, 50 cases, 24 males and 26 females, were normal children and examined in hospitals. There was no statistically significant difference in general data between the two groups (*p* > 0.05).

### 2.4 qPCR detected the 10 core genes in sepsis

Extraction of serum RNA: Serum tRNA extraction kit (mirVanaPARIS Kit; AM1556) Ambion, USA. The main steps were as follows: Take 350 μl of serum and perform total RNA extraction according to the kit instructions. Total RNA was dissolved in 50 μl Ambion RNA eluate, and the final amount was about 45 μl. Serum total RNA quality testing primarily excludes genomic DNA contamination. The total RNA extracted from 350 μl of serum was approximately 500 ng, and any sample larger than 500 ng in total will be rejected. In order to eliminate genomic DNA contamination, gradient genomic DNA was added as a positive control in the qRT‐PCR experiment, and the first strand was reverse‐transcribed to synthesize cDNA. Design‐specific primers according to the prime sequence of these genes (Table 1) optimize the optimal PCR conditions, use SYBR Green I RT‐PCR to amplify the target fragment, and detect the fluorescence intensity of the product in real time. The internal reference uses the human β‐actin gene. The relative content of the sample to be tested was calculated according to the 2^−ΔΔCT^ formula.^7^ The study met the medical ethics standards and was approved by ethics committee of the First Affiliated Hospital of Wenzhou Medical University. All treatments and examinations were informed by the patient or family.

**Table 1:** Primer sequence of core genes

Gene name	Sense 5′‐3′	Antisense 5′‐3′	Product length (bp)
HCK	GCAACACACCAGGAATCAGG	CCCGGATTCCTCTAGGACCA	134
PRKCD	TGGTGGTTGGTGCGTTGTAG	TTTTCCCACGCTCTGTGCT	214
SIRPA	AAATACCGCCGCTGAGAACA	TGTGATATCATTTGTGTCCTGTGT	200
DOK3	CAGCACAGTGGTCTTGAAGC	CTGATGGGCCTCCTGCATAC	160
ITGAM	TAACATCACCAACGGAGCCC	CTCCCACCCCAATGACGTAG	149
LTB4R	CTCTACGTCTTCACCGCTGG	GGAGGACCTCTGGAGGGTG	189
MAPK14	CAGCTTCAGCAGATTATGCGTC	AGTCGACAGCCAGGGGATTG	160
MALT1	AGTGGAGTGCACTGAAGATGAA	AAGGAGCTTTGAGCTTGGGG	146
NLRC3	AGGATCTCACAATCTCAGGGAC	TCATCCCTAACGGTGTTGCC	202
LCK	AGATCAGCTTGGCGGAGAAC	CACCTCAGAGCCATTTCGGA	155
B‐actin	ACAGAGCCTCGCCTTTGC	CCACCATCACGCCCTGG	191

### 2.5 Statistical analysis

Statistical analysis was performed by SPSS software v19 (SPSS Inc, USA). The data of each group were tested for normality and homogeneity of variance first. The data that accorded with normal distribution and homogeneity of variance were analyzed by one‐way analysis of variance. The data that did not conform to normal distribution and homogeneity of variance were analyzed by nonparametric analysis. The normal distribution data were represented by “average standard ± deviation,” and the non‐normal distribution data were represented by the median (interquartile range). The receiver operating characteristic (ROC) curve was then used to estimate the classification performance. Differences were considered statistically significant with *p *< 0.05.

## 3 RESULTS

### 3.1 Differentially expressed genes in pediatric sepsis and normal group

First, the GSE13904 dataset (including 209 pediatric sepsis samples and 18 normal samples), GSE25504 dataset (including 44 pediatric sepsis samples and 44 normal samples), and GSE26440 dataset (including 98 pediatric sepsis samples and 32 normal samples) were used to express the spectral data, the probes were compared to the gene symbol using the platform annotation file, and the differentially expressed genes were screened using the Rlimma package. According to the multiple of difference (|logFC| > 0.585) and significance threshold (FDR < 0.05), a total of 758 differentially expressed genes were screened using R package limma, of which 580 were up‐regulated and 178 were down‐regulated and shown in Table S1 and Figure S1.

### 3.2 WGCNA co‐expression analysis

**Figure 2:**
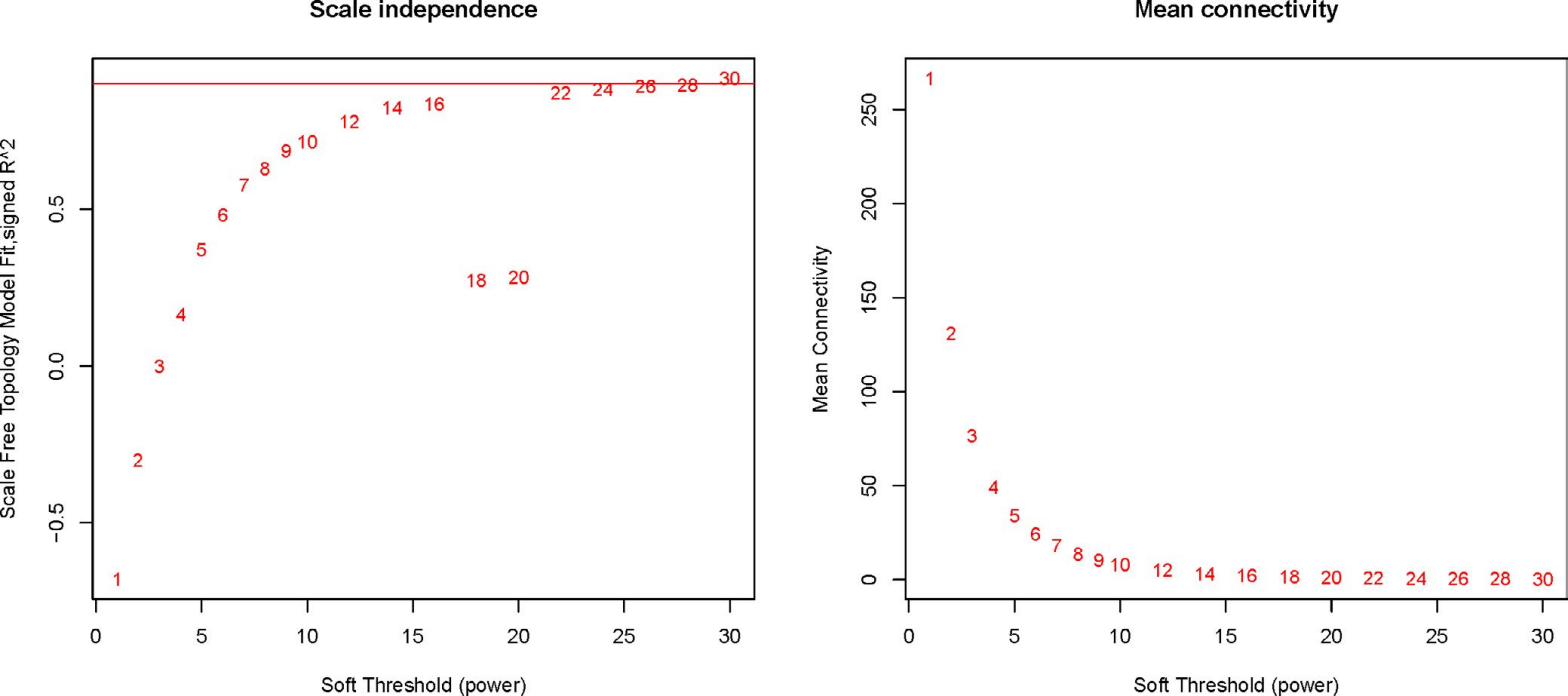
Optimal threshold selection map for pediatric sepsis WGCNA analysis. 758 potential differentially expressed gene expression profiles were constructed and analyzed for WGCNA co‐expression using the R package (minimum module size set to 30, merge Cut Height = 0.25, verbose = 3). The optimal threshold for WGCNA analysis of potential gene expression profiles for analysis was 22

First, the differentially expressed genes of three sets of pediatric sepsis expression profiles (GSE13904, GSE25504, GSE26440) were pooled and a total of 758 potentially differentially expressed genes were obtained. Using the GSE13904 dataset, 758 potential differentially expressed gene expression profiles were constructed and analyzed for WGCNA co‐expression using the R package (minimum module size set to 30, merge Cut Height = 0.25, verbose = 3). The optimal threshold for WGCNA analysis of potential gene expression profiles for analysis was 22, as shown in Figure 2.

**Figure 3:**
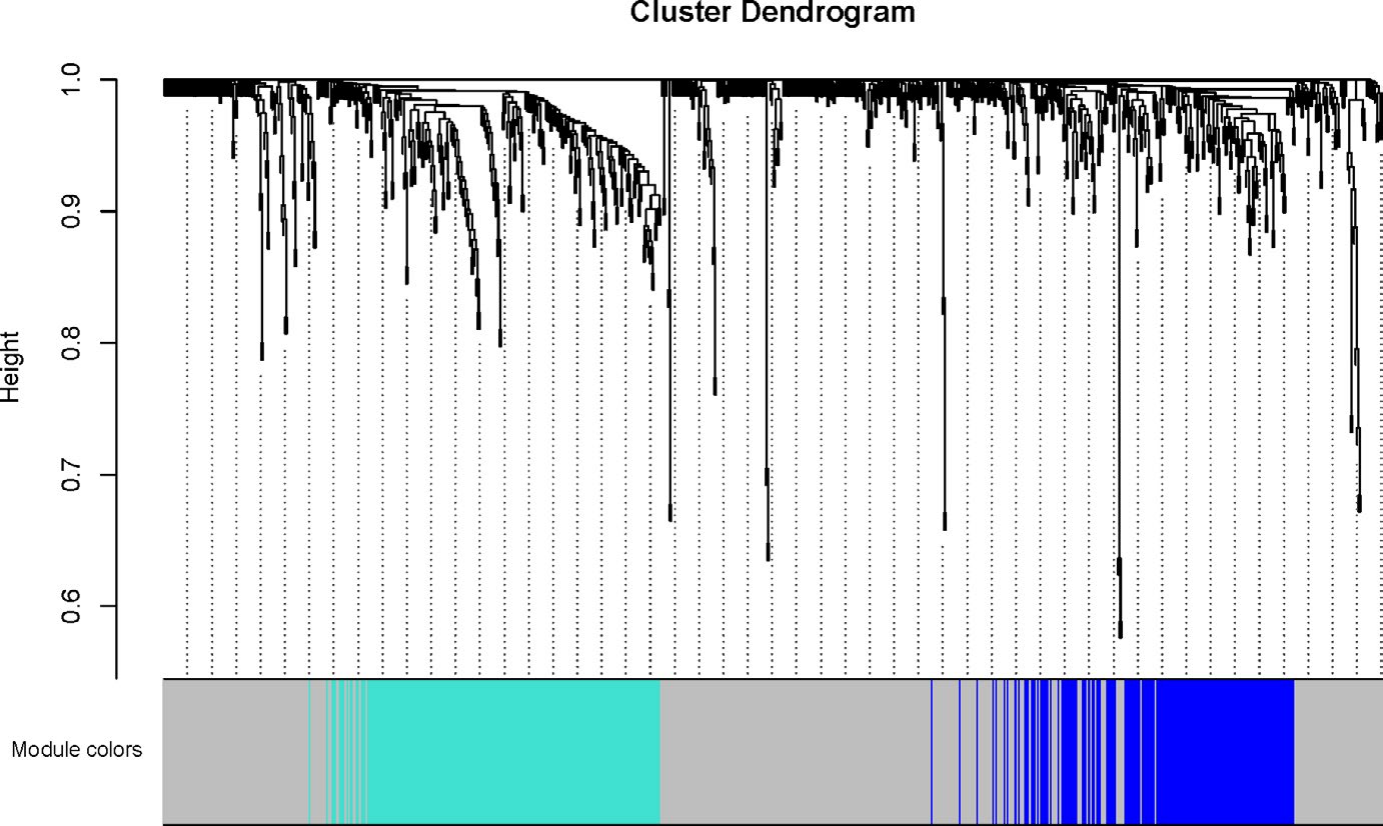
Pediatric sepsis‐related module clustering system tree diagram. A total of 3 modules were mined in the final gene expression profile of pediatric sepsis

**Figure 4:**
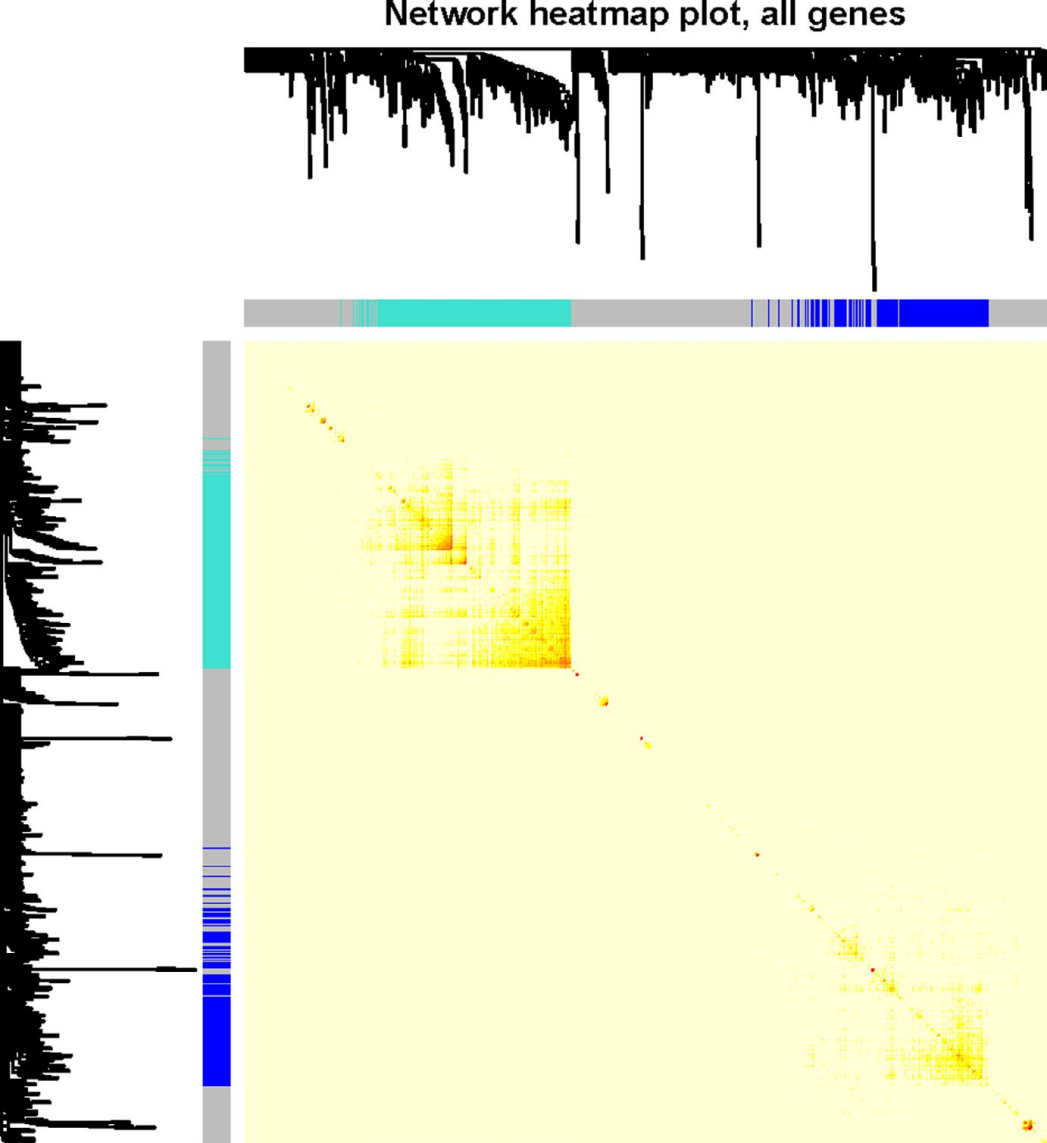
Pediatric sepsis module co‐expressed cluster heat map. Co‐expression results revealed the presence of multiple pediatric sepsis‐associated genes

**Figure 5:**
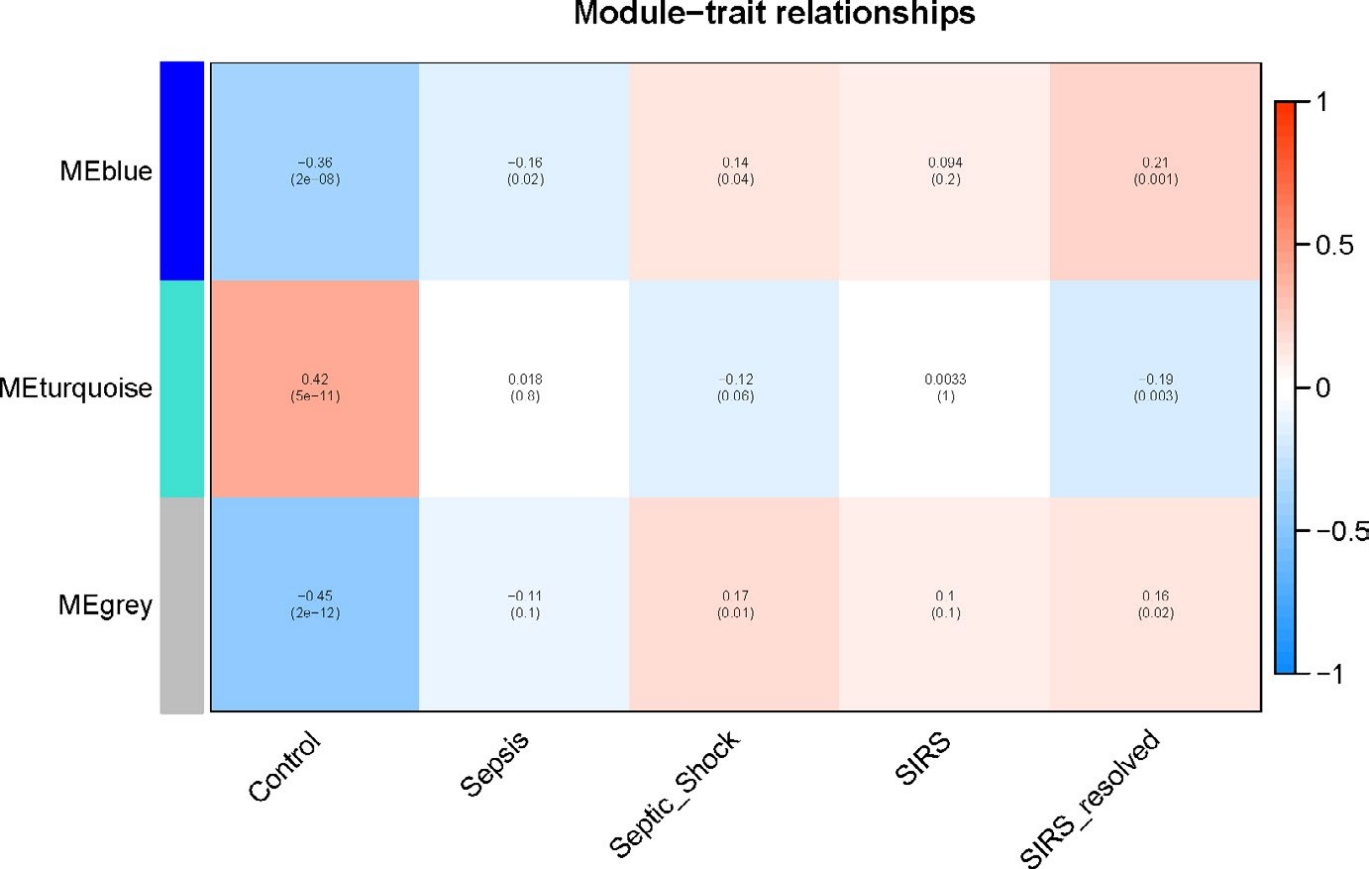
Relationship between pediatric sepsis module and traits. The gene expression level in the blue module was significantly negatively correlated with normal features, significantly negatively correlated with the pediatric sepsis feature, and significantly positive with the Septic shock feature. Correlation was significantly positively correlated with SIRS resolved features. Gene expression in the turquoise module was significantly positively correlated with normal features and significantly negatively correlated with SIRS resolved features. The genes in the gray module were genes that were not assigned to the module

**Figure 6:**
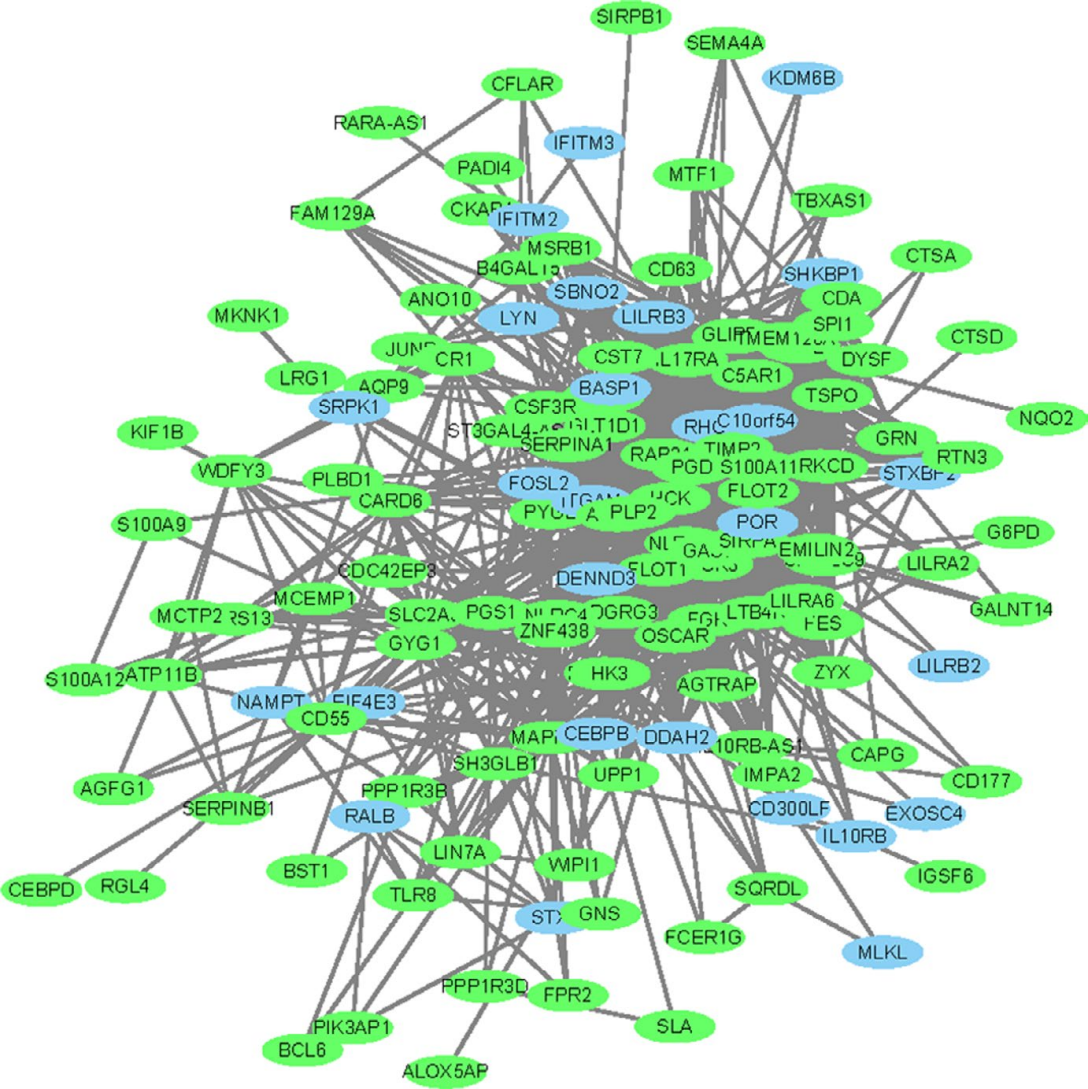
Pediatric sepsis blue module network diagram. A network diagram of the pediatric sepsis blue module showing the presence of multiple genes up‐regulated or down‐regulated

In the final potential gene expression profile of analysis, three modules were mined. Figure 3 was a systematic tree diagram of module clustering. The heat map of co‐expression analysis was shown in Figure 4. It shown three analysis modules and traits (The relationship between analysis, Septic Shock, SIRS, SIRS resolved, normal) and the gene expression level in the blue module was significantly negatively correlated with normal features, significantly negatively correlated with the pediatric sepsis feature, and significantly positive with the Septic shock feature from Figure 6. Correlation was significantly positively correlated with SIRS resolved features. Gene expression in the turquoise module was positively correlated with normal features and negatively correlated with SIRS resolved features. The genes in the gray module were genes that were not assigned to the module (Figure 5).

### 3.3 Module network construction and hub gene screening

**Figure 7:**
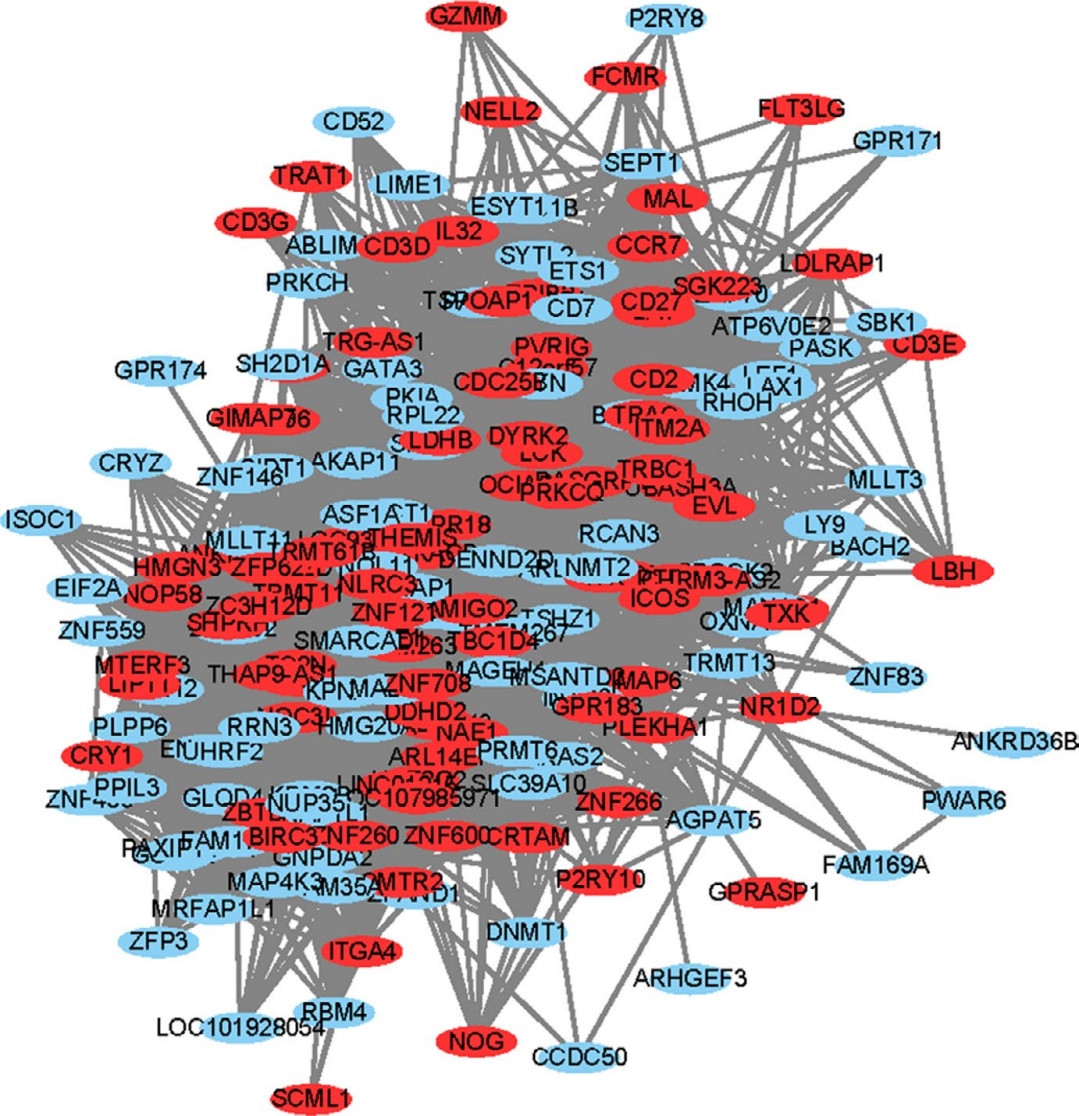
Pediatric sepsis turquoise module network diagram. A network map of the pediatric sepsis turquoise module (green for down‐regulated genes, red for up‐regulated genes), showing the presence of multiple genes up‐regulated or down‐regulated

The cytoscape was used to construct the network of key pediatric sepsis modules. Figure 6 was the network diagram of the pediatric sepsis blue module, and Figure 7 was the network diagram of the pediatric sepsis turquoise module (green represents the down‐regulated gene and red represents the up‐regulated gene). The degree distribution of two pediatric sepsis critical modules (blue, turquoise) networks was analyzed, and the top 20 gene was selected as the pediatric sepsis hub gene for subsequent analysis, included TIMP2, FLOT1, HCK, NCF4, SERPINA1, IL17RA, PGD, PRKCD, GLT1D1, ALOX5, SIRPA, DOK3, ITGAM, S100A11, ZNF438, PLIN3, LTB4R, TSPO, MAPK14, GAS7 and TBC1D4, NOL11, NLRC3, ZNF121, DYRK2, ABCE1, MAGEH1, TMEM263, MCUB, MALT1, DDHD2, TRAC, NOC3L, LCK, TRMT61B, ZNF260, ENOPH1, LOC93622, NAE1, TRBC1.

### 3.4 Function and pathway enrichment analysis of key modules of pediatric sepsis

EnrichR was used to analyze the function and pathway enrichment of the medium top20 genes in two pediatric sepsis critical modules (blue, turquoise). The GO function enrichment results of the blue module are shown in Figure S2A,B,C. The KEGG pathway enrichment results were shown in Figure S3. The GO function enrichment results of the turquoise module are shown in Figure S4A,B,C. The KEGG pathway enrichment results are shown in Figure S5.

From the results of enrichment analysis, the top 20 genes of the blue module of pediatric sepsis were mainly enriched in neutrophil degranulation, neutrophil‐mediated immunity, fibrin‐1‐rich particle cavity, cysteine. Glytriene receptor activity, leukotriene receptor activity, protease binding and other GO term enriched in amyotrophic lateral sclerosis, arachidonic acid metabolism, complement and coagulation cascade, Salmonella infection, RIG‐I‐like receptor signaling pathway, acute myeloid leukemia, toxoplasmosis, IL‐17 signaling pathway and other KEGG pathways. The top 20 genes for the pediatric sepsis turquoise module were mainly enriched in rRNA‐containing ribonucleoprotein complexes exported from the nucleus, NIK/NF‐kappaB signal negative regulation, T‐cell receptor signaling pathway, centromeric peripheral material, mitochondrial inner membrane GO term, CD4 receptor binding, phosphatidylinositol 3‐kinase regulatory subunit binding, etc, enriched in NF‐kappa B signaling pathway, cysteine and methionine metabolism, Th17 cell differentiation, T‐cell receptor signal path and other KEGG pathways.

### 3.5 Verification of pediatric sepsis biomarkers

**Figure 8:**
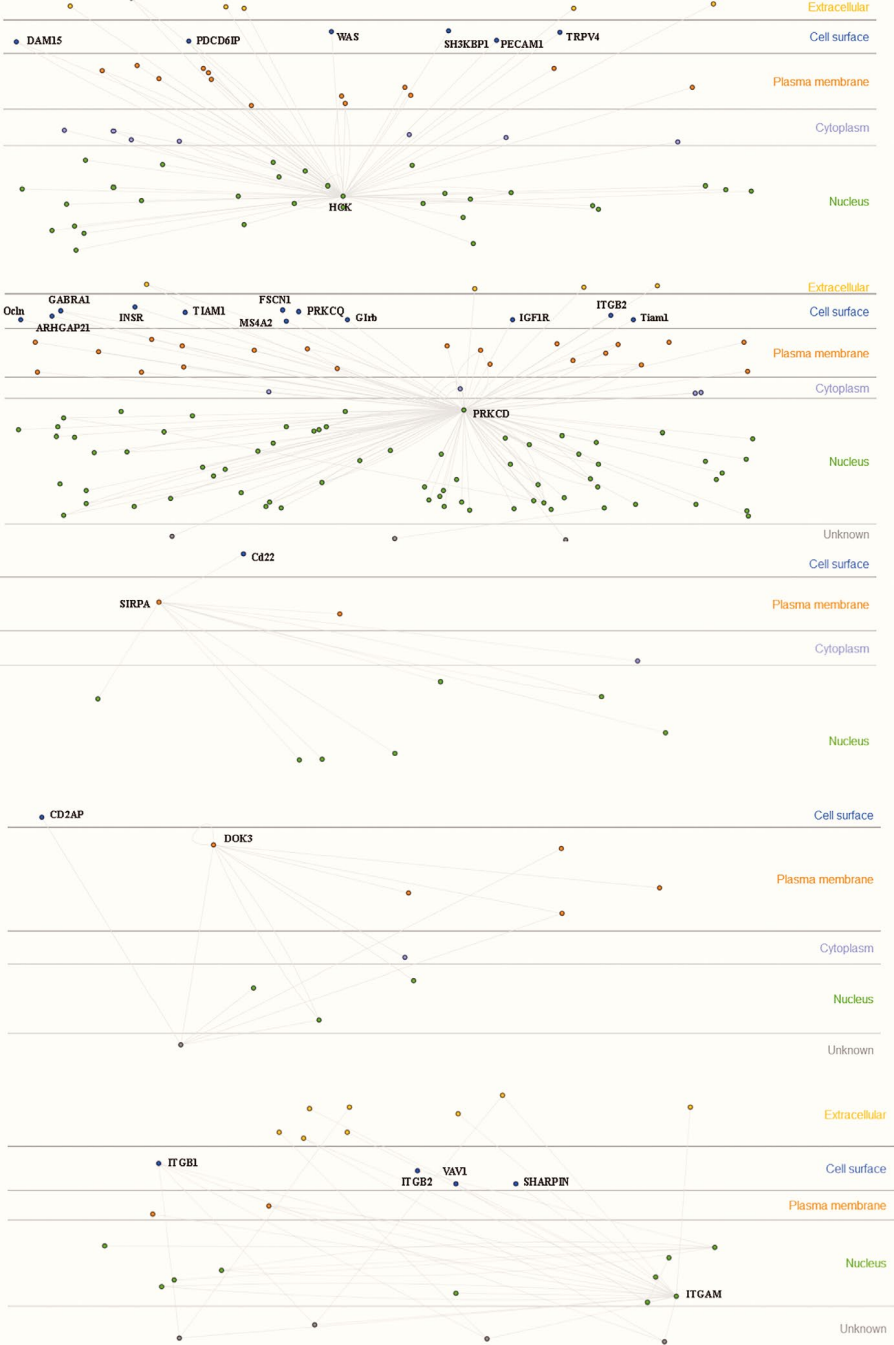
Pediatric sepsis 5 core genes experimental verification interaction. It shown that HCK, PRKCD, SIRPA, DOK3, ITGAM genes were interacted with molecules related to cell surface, plasma membrane, nucleus, and cytoplasm from the Innate DB database

**Figure 9:**
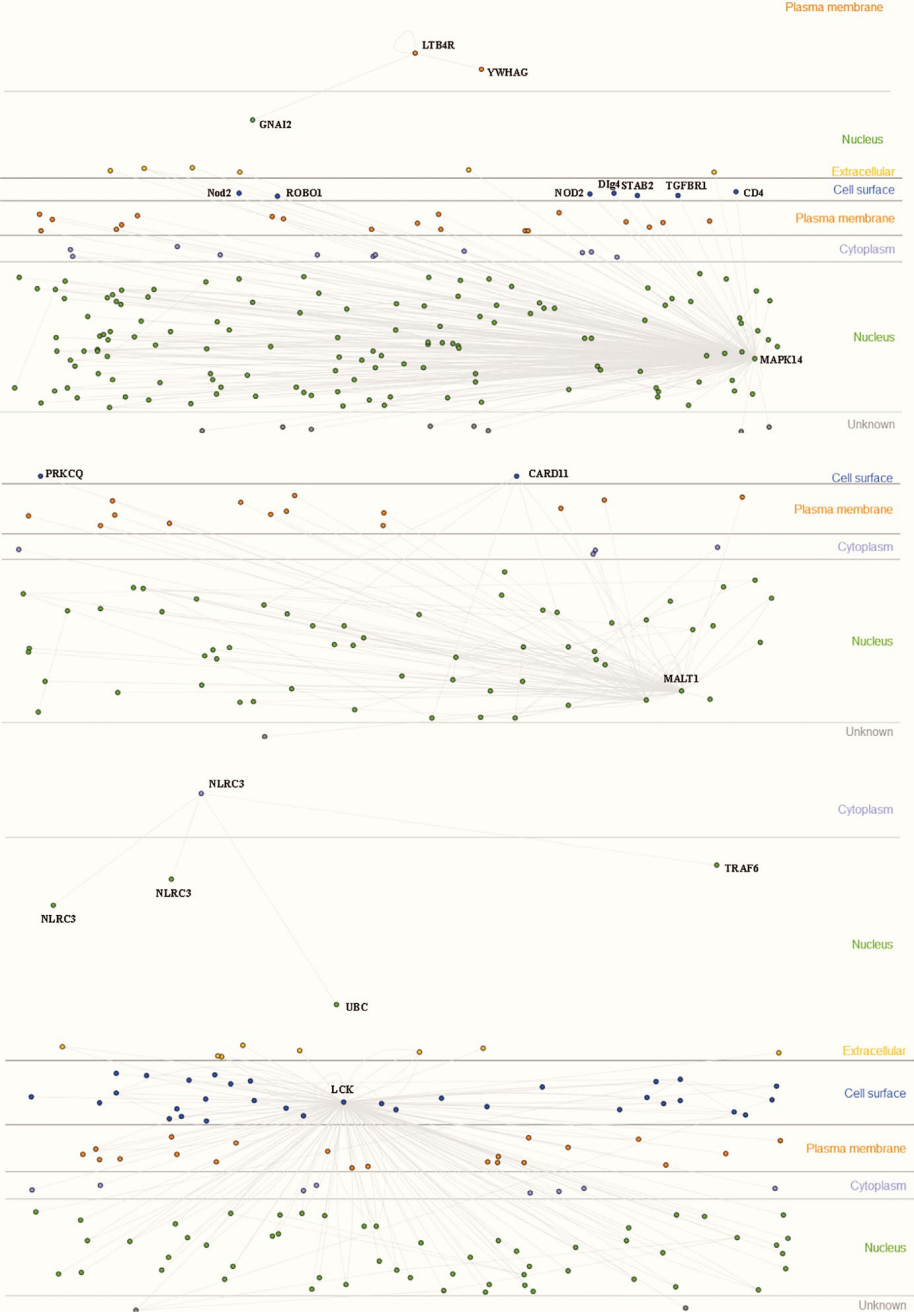
Pediatric sepsis 5 core genes experimental verification interaction. It shown that LTB4R, MAPK14, MALT1, NLRC3, LCK genes were interacted with molecules related to cell surface, plasma membrane, nucleus, and cytoplasm from the Innate DB database

Using the Innate DB database, the selected pediatric sepsis hub gene was combined with the marker on the cell surface to verify, and the validated marker was selected as the characteristic gene (core genes) for constructing the panel.^8^ A total of 10 core genes (HCK, PRKCD, SIRPA, DOK3, ITGAM, LTB4R, MAPK14, MALT1, NLRC3, LCK) were obtained. The experimental validation interactions of HCK, PRKCD, SIRPA, DOK3, ITGAM genes were shown in Figure 8 and LTB4R, MAPK14, MALT1, NLRC3, LCK genes were shown in Figure 9.

### 3.6 Construction and verification of pediatric sepsis diagnostic panel

**Figure 10:**
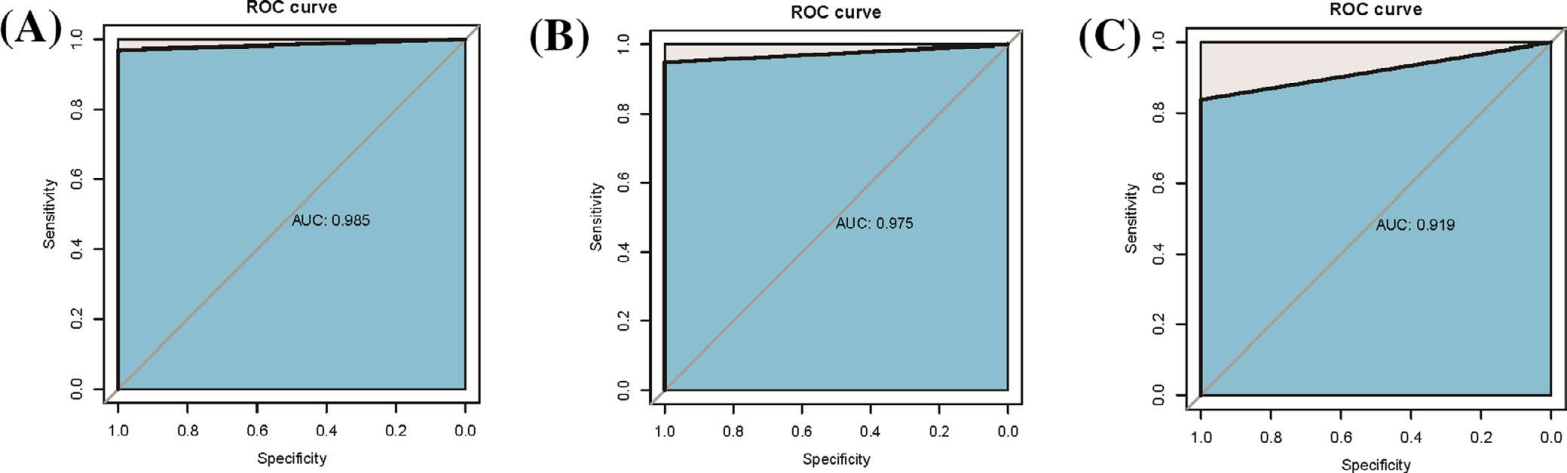
ROC curve of syringe GSE13904 training set classification, test set classification and GSE26440. (A) ROC curve obtained by using the elastic network panel in the training set and the area under the curve was 0.985. (B) The ROC curve obtained by using the elastic network panel in the test and the area under the curve was 0. 975. (C) Validation was performed using the independent pediatric sepsis sample set GSE26440 and were a under the curve was 0.919

Using the selected 10 core genes panel (HCK, PRKCD, SIRPA, DOK3, ITGAM, LTB4R, MAPK14, MALT1, NLRC3, LCK) as a panel features, the GSE13904 sample set was randomly divided into training set and test set according to 2:1. A pediatric sepsis diagnostic panel was constructed using an elastic regression network (R package glmnet) to screen the main classification features and construct a diagnostic model. Finally, the ROC curve obtained by using the elastic network panel in the training set was shown in Figure 10A, and the area under the curve was 0.985. The ROC curve obtained by using the elastic network panel in the test was shown in Figure 10B, and the area under the curve was 0.975.

Finally, validation was performed using the independent pediatric sepsis sample set GSE26440. The resulting ROC curve was shown in Figure 10C, with an area under the curve of 0.919. From the results, it can be found that using the elastic network panel model, the selected 10 core genes (HCK, PRKCD, SIRPA, DOK3, ITGAM, LTB4R, MAPK14, MALT1, NLRC3, LCK) were used as a panel, and the independent sample set was used for verification. The area under the ROC curve was above 0.9, indicating that the panel has high accuracy of diagnosis for pediatric sepsis.

### 3.7 The result of clinical sample verification

**Figure 11:**
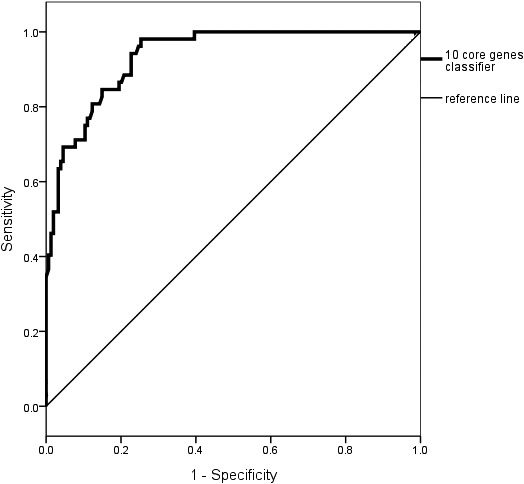
ROC curve of diagnosis of pediatric sepsis. The AUC of 10 core genes panel was 0.936 in diagnosis of pediatric sepsis

According to ROC figure, the AUC of 10 core genes panel (0.936), sensitivity (96.6%), and specificity (58.7%) was in diagnosis of pediatric sepsis (Figure 11 and Table 2). Our results showed that the clinical outcomes of pediatric sepsis patients with 10 core genes panel were consistent with bioinformatic predictions, suggesting that the 10 core genes panel was expected to be a good biomarker spectrum for the diagnosis of pediatric sepsis.

**Table 2:** The AUC, cutoff value, sensitivity, and specificity of parameters for the diagnosis of sepsis

Parameters	AUC	95% confidence Interval	Cutoff	Sensitivity (%)	Specificity (%)
10 core genes panel	0.936	0.904–0.968	14.363	96.6	58.7

## 4 DISCUSSION

Sepsis is a common critical illness, and its high morbidity and mortality had attracted the attention of critical medical experts. The mortality rate of sepsis patients in this study was 26.11%, which was consistent with the mortality rate of sepsis in domestic and foreign literature.^9^, ^10^ In order to more effectively assess the prognosis of sepsis, scholars continue to explore more simple and effective monitoring indicators and methods. The APACHE II scoring system is one of the most commonly used criticality assessment systems for patients with clinical sepsis. The higher the score, the more critical the condition and the worse the prognosis.^11^

Taken together, it was difficult to achieve an accurate and comprehensive assessment of the prognostic indicators of a single sepsis, and recent studies had shown that multiple indicators need to be combined to evaluate.^12^ Due to the lack of a genetic database for sepsis in adults, we tried to use the genetic database of pediatric sepsis to obtain some differential genes, and further verified it in clinical samples of adult sepsis.

Our findings showed that differential gene of pediatric sepsis was mainly enriched in neutrophil degranulation, neutrophil‐mediated immunity, fibrin‐1‐rich particle cavity, cysteine, rRNA‐containing ribonucleoprotein complexes exported from the nucleus, NIK/NF‐kappaB signal negative regulation, T‐cell receptor signaling pathway, and so on. These studies show that the above pathway might play an important role in pediatric sepsis.

The selected hub gene of pediatric sepsis was combined with the markers of cell surface and found 10 core genes (HCK, PRKCD, SIRPA, DOK3, ITGAM, LTB4R, MAPK14, MALT1, NLRC3, LCK) by downloading data and bioinformatics analysis of childhood sepsis. ROC analysis showed that AUC of the 10 core genes for diagnosis of pediatric sepsis was above 0.9. This result showed 10 core genes had a high diagnostic efficiency for pediatric sepsis.

In order to further verify the clinical value of 10 core genes, we found that the AUC of 10 core genes panel was 0.936 in diagnosis of pediatric sepsis. It helped clinicians to accurately identify the prognosis and diagnosis of pediatric sepsis in the early stage, optimize the management of pediatric sepsis treatment as soon as possible, and minimize mortality rate of the patients with pediatric sepsis.

## 5 CONCLUSIONS

In conclusion, there were many abnormally expressed genes in patients with pediatric sepsis. The panel constructed by the 10 core genes was very effective in diagnosing of pediatric sepsis and was expected to become a biomarker panel for clinical application.

## CONFLICT OF INTERESTS

The authors declare that they have no competing interests.

## Supporting information

Figure S1

Figure S2

Figure S3

Figure S4

Figure S5

Table S1

## Data Availability

The data that support the findings of this study are available in GEO database: GSE13904 (GPL570 platform), GSE25504 (GPL570, GPL6947, GPL13667 platform), GSE26440 (GPL570 platform) and these data were derived from the GEO database.
