# Supplementary figures and images for: Constructing a 10‐core genes panel for diagnosis of pediatric sepsis
Source: J Clin Lab Anal. 2020 Dec 3;35(3):e23680. doi: 10.1002/jcla.23680 (PMC7958006; doi:10.1002/jcla.23680)

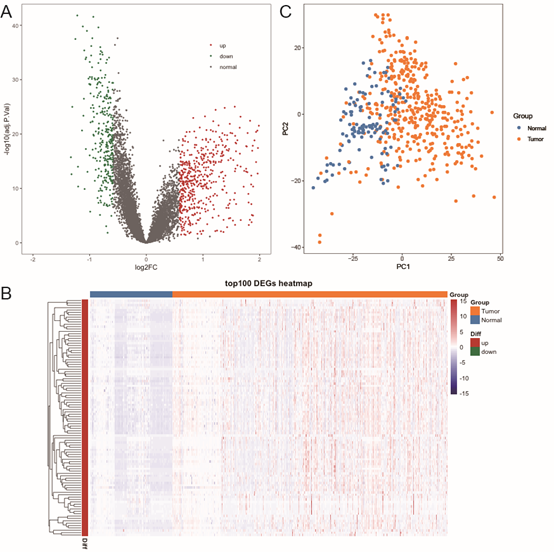

Supplement: Supplementary file 1 — Figure S1 [file JCLA-35-e23680-s006.tif]

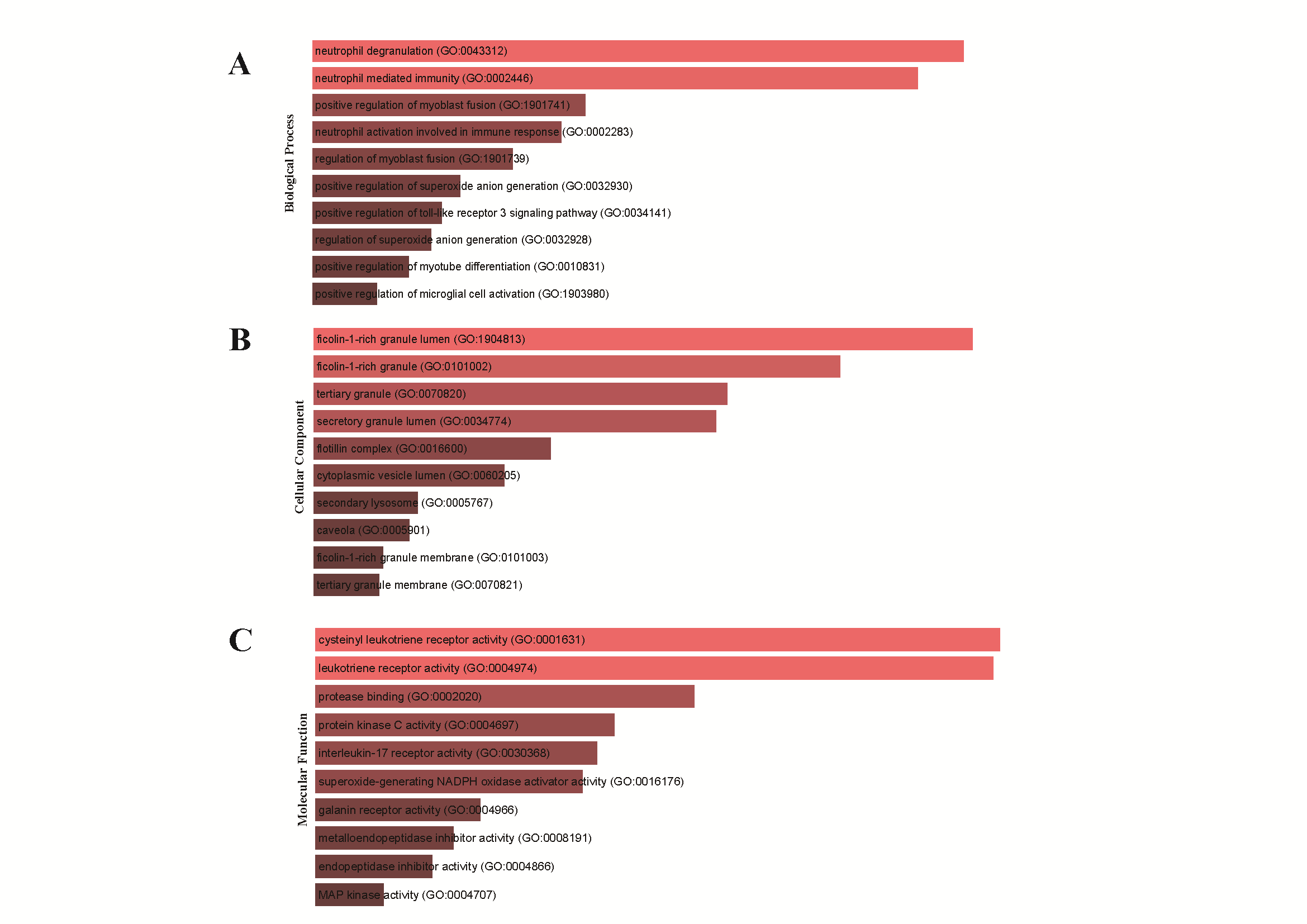

Supplement: Supplementary file 2 — Figure S2 [file JCLA-35-e23680-s003.tif]

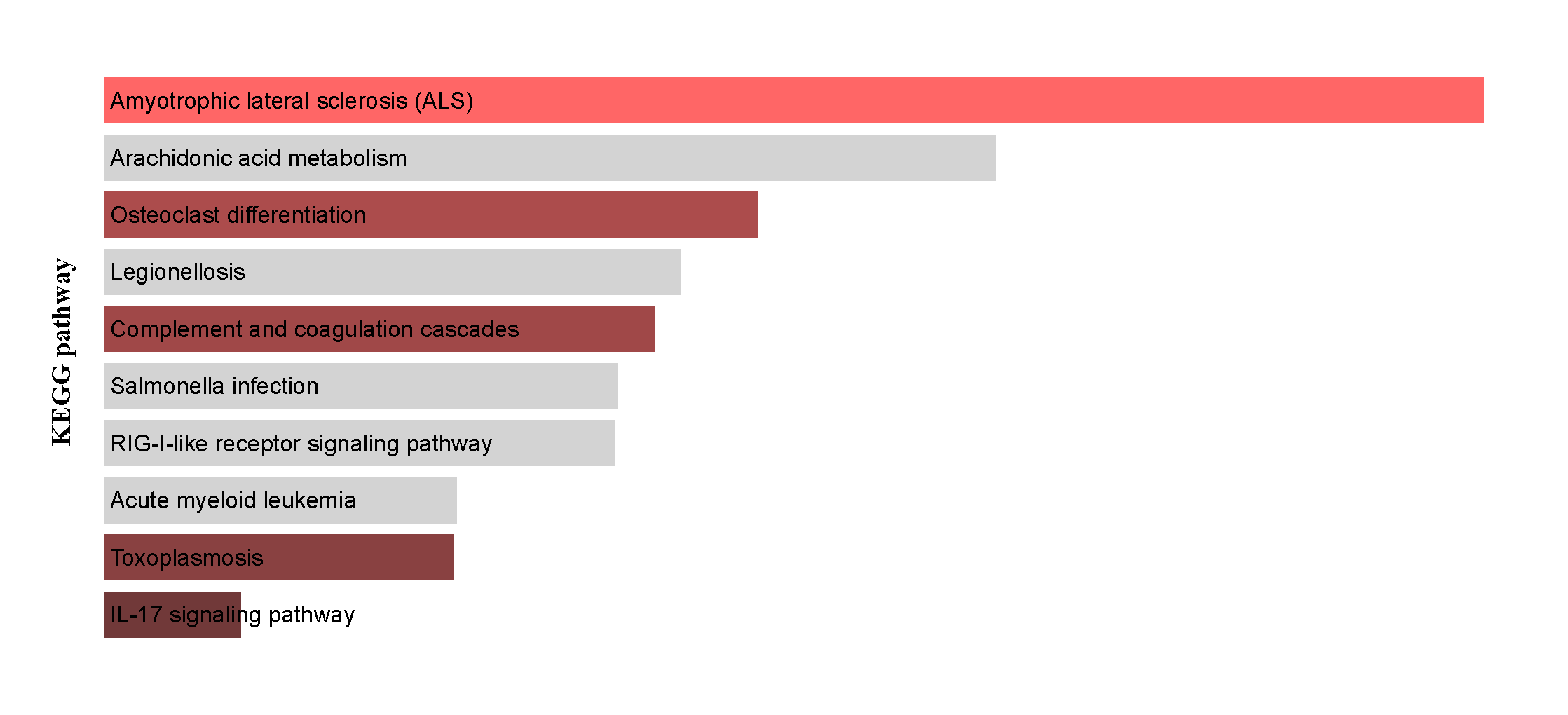

Supplement: Supplementary file 3 — Figure S3 [file JCLA-35-e23680-s002.tif]

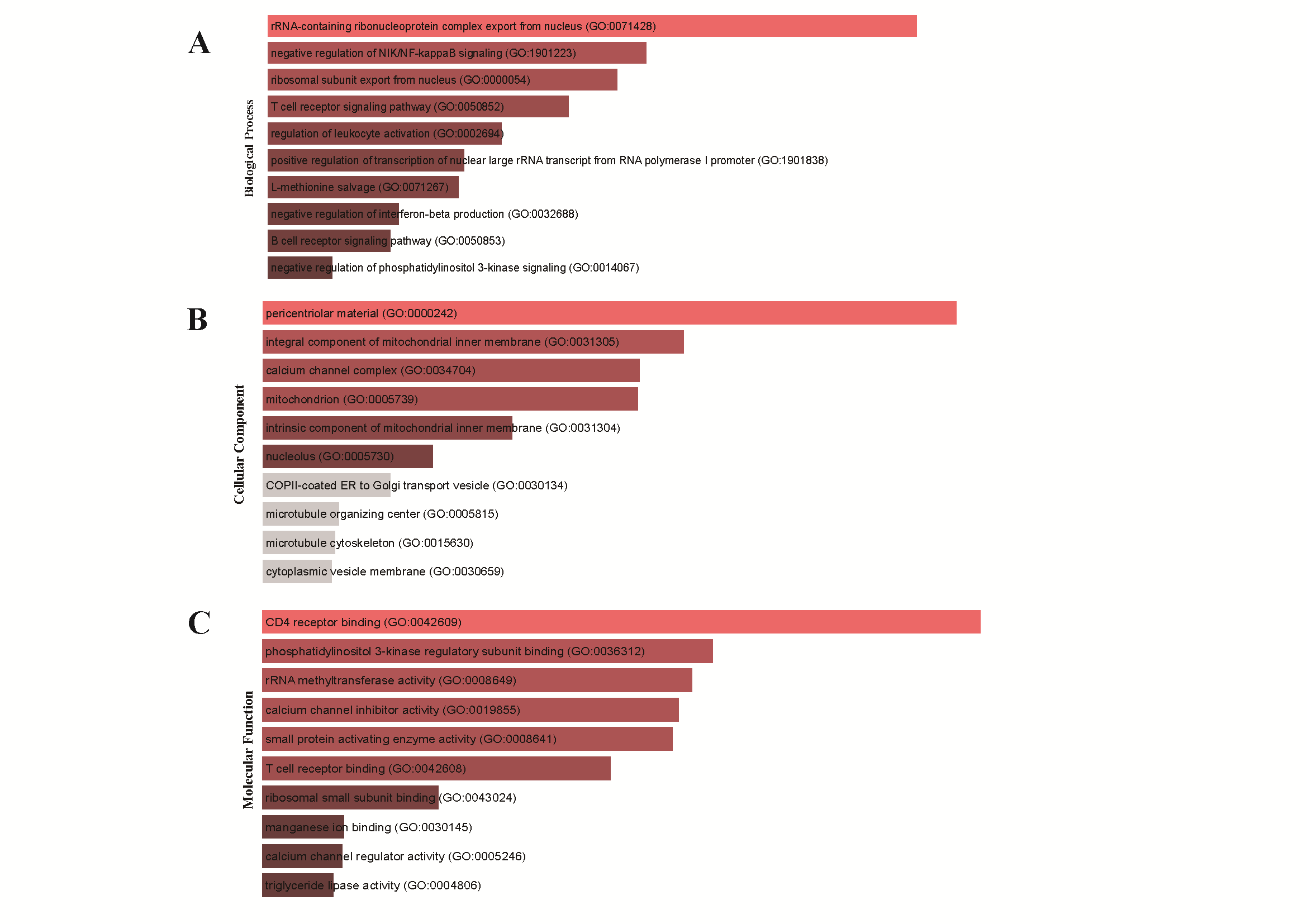

Supplement: Supplementary file 4 — Figure S4 [file JCLA-35-e23680-s004.tif]

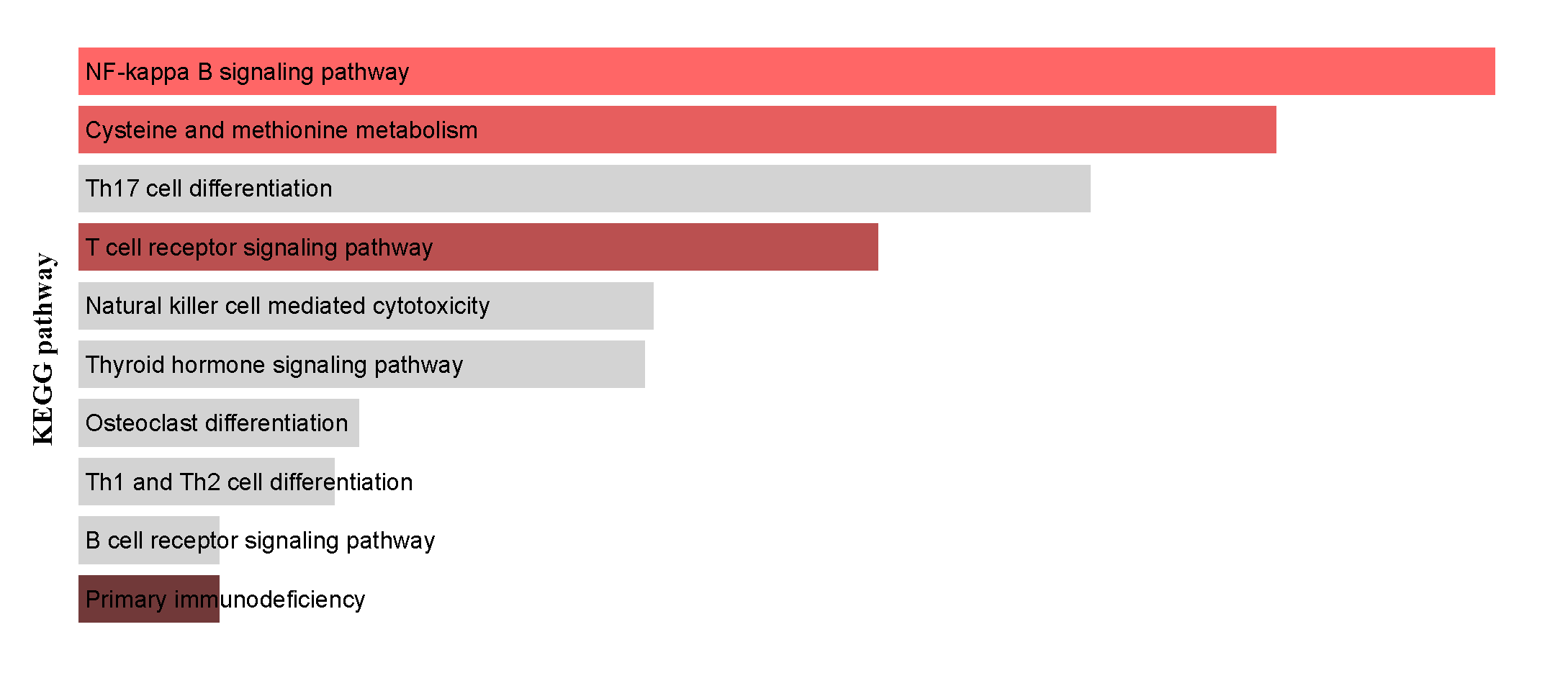

Supplement: Supplementary file 5 — Figure S5 [file JCLA-35-e23680-s005.tif]
