# Supplementary material for: Constructing a 10‐core genes panel for diagnosis of pediatric sepsis
Source: J Clin Lab Anal. 2020 Dec 3;35(3):e23680. doi: 10.1002/jcla.23680 (PMC7958006; doi:10.1002/jcla.23680)
Supplement: Supplementary file 6 — Table S1 [file JCLA-35-e23680-s001.docx]

| gene | logFC | AveExpr | t | P.Value | adj.P.Val | B |
| --- | --- | --- | --- | --- | --- | --- |
| MMP8 | 64.19467 | 96.52635 | 5.189153 | 3.02E-07 | 1.06E-06 | 5.953043 |
| OLFM4 | 44.1597 | 65.41121 | 4.232214 | 2.73E-05 | 7.22E-05 | 1.649509 |
| CD177 | 22.75811 | 34.06745 | 6.729887 | 4.43E-11 | 2.65E-10 | 14.50553 |
| LTF | 12.91234 | 20.38784 | 5.767441 | 1.37E-08 | 5.84E-08 | 8.935622 |
| LCN2 | 12.74319 | 17.05889 | 6.282709 | 6.96E-10 | 3.58E-09 | 11.82573 |
| MMP9 | 11.97473 | 16.32046 | 9.079544 | 2.16E-18 | 4.10E-17 | 30.99328 |
| RETN | 11.56514 | 15.20613 | 7.465524 | 3.44E-13 | 2.82E-12 | 19.24678 |
| HP | 10.62398 | 14.89653 | 7.44847 | 3.87E-13 | 3.14E-12 | 19.13232 |
| ANXA3 | 10.59551 | 16.68176 | 9.657054 | 1.98E-20 | 5.12E-19 | 35.6064 |
| OLAH | 9.891675 | 15.9413 | 4.411649 | 1.24E-05 | 3.47E-05 | 2.394936 |
| IL1R2 | 9.5177 | 14.51248 | 8.194344 | 1.91E-15 | 2.20E-14 | 24.33203 |
| GPR84 | 8.322929 | 10.93054 | 9.009239 | 3.77E-18 | 6.89E-17 | 30.4457 |
| ANKRD22 | 7.889512 | 10.37088 | 7.974471 | 9.53E-15 | 9.90E-14 | 22.75864 |
| DEFA4 | 7.231802 | 12.9134 | 4.042111 | 6.08E-05 | 0.000152 | 0.891346 |
| CLEC5A | 6.226571 | 9.43775 | 7.005349 | 7.53E-12 | 4.95E-11 | 16.23332 |
| VNN1 | 5.912068 | 9.41321 | 8.562919 | 1.21E-16 | 1.64E-15 | 27.04317 |
| TCN1 | 5.566474 | 9.364084 | 6.282067 | 6.99E-10 | 3.59E-09 | 11.822 |
| IFI27 | 5.56019 | 15.41309 | 2.351068 | 0.019086 | 0.03102 | -4.37992 |
| CRISP3 | 5.342007 | 10.38668 | 4.210014 | 3.00E-05 | 7.88E-05 | 1.559288 |
| IL18R1 | 4.794186 | 7.33415 | 7.851269 | 2.31E-14 | 2.25E-13 | 21.89167 |
| MS4A4A | 4.765788 | 9.292855 | 5.539838 | 4.78E-08 | 1.88E-07 | 7.728269 |
| ARG1 | 4.687964 | 7.920822 | 8.97764 | 4.84E-18 | 8.56E-17 | 30.20061 |
| CLEC4D | 4.548816 | 7.361784 | 8.812285 | 1.77E-17 | 2.82E-16 | 28.92839 |
| PRTN3 | 4.471046 | 5.831891 | 4.828698 | 1.80E-06 | 5.70E-06 | 4.237881 |
| BMX | 4.30672 | 7.648219 | 8.630603 | 7.19E-17 | 1.03E-15 | 27.55085 |
| GYG1 | 4.151699 | 7.115528 | 11.32695 | 8.90E-27 | 7.08E-25 | 50.00879 |
| AZU1 | 3.96255 | 6.33635 | 4.361043 | 1.56E-05 | 4.28E-05 | 2.181784 |
| FOLR3 | 3.954103 | 8.148432 | 3.959434 | 8.54E-05 | 0.000208 | 0.57183 |
| ALPL | 3.947228 | 7.372007 | 8.151744 | 2.62E-15 | 2.94E-14 | 24.0246 |
| ORM1 | 3.895584 | 7.15286 | 6.378366 | 3.91E-10 | 2.09E-09 | 12.38576 |
| PFKFB3 | 3.886757 | 6.900699 | 10.17467 | 2.51E-22 | 9.35E-21 | 39.90857 |
| CA4 | 3.855074 | 6.909742 | 8.985056 | 4.57E-18 | 8.15E-17 | 30.25807 |
| MPO | 3.831662 | 5.804455 | 4.860118 | 1.55E-06 | 4.97E-06 | 4.382912 |
| CEACAM6 | 3.824337 | 6.213913 | 3.702988 | 0.000235 | 0.000536 | -0.37955 |
| PGLYRP1 | 3.812018 | 6.967615 | 8.714046 | 3.79E-17 | 5.73E-16 | 28.18087 |
| RGL4 | 3.751227 | 6.62247 | 8.675279 | 5.10E-17 | 7.52E-16 | 27.88761 |
| CD163 | 3.5383 | 6.684539 | 6.02815 | 3.11E-09 | 1.47E-08 | 10.37103 |
| CEACAM1 | 3.525494 | 6.116902 | 9.640216 | 2.28E-20 | 5.81E-19 | 35.46906 |
| CYP1B1 | 3.413022 | 7.087707 | 6.960733 | 1.01E-11 | 6.53E-11 | 15.94956 |
| MGAM | 3.365698 | 6.587008 | 11.34894 | 7.27E-27 | 5.94E-25 | 50.20811 |
| MS4A3 | 3.361519 | 6.741387 | 3.463225 | 0.000577 | 0.00124 | -1.21428 |
| IRAK3 | 3.296229 | 6.439091 | 10.72687 | 2.01E-24 | 1.08E-22 | 44.66252 |
| S100A12 | 3.20606 | 7.790186 | 13.31124 | 4.53E-35 | 1.58E-32 | 68.85898 |
| HK3 | 3.140309 | 6.348489 | 10.62988 | 4.76E-24 | 2.40E-22 | 43.81569 |
| FCGR1A | 3.097069 | 6.144806 | 8.828917 | 1.55E-17 | 2.52E-16 | 29.05557 |
| DYSF | 3.057796 | 6.787663 | 11.9721 | 2.16E-29 | 2.60E-27 | 55.95105 |
| LRG1 | 2.985784 | 6.28669 | 10.39097 | 3.87E-23 | 1.68E-21 | 41.75108 |
| GADD45A | 2.819304 | 6.422937 | 8.953521 | 5.86E-18 | 1.02E-16 | 30.01396 |
| TLR5 | 2.797825 | 5.889078 | 10.66 | 3.65E-24 | 1.88E-22 | 44.07811 |
| BCL2A1 | 2.73626 | 5.92352 | 8.576232 | 1.09E-16 | 1.49E-15 | 27.14279 |
| DHRS9 | 2.717543 | 5.66113 | 6.9895 | 8.35E-12 | 5.45E-11 | 16.13235 |
| FGF13 | 2.654881 | 6.009992 | 3.808455 | 0.000156 | 0.000366 | 0.004425 |
| TNFAIP6 | 2.587647 | 6.035062 | 8.454879 | 2.74E-16 | 3.54E-15 | 26.23904 |
| PSTPIP2 | 2.582988 | 5.46492 | 10.16979 | 2.62E-22 | 9.71E-21 | 39.86729 |
| ADM | 2.514677 | 5.800791 | 9.876793 | 3.16E-21 | 9.43E-20 | 37.41399 |
| C1QB | 2.506379 | 4.781117 | 4.630673 | 4.59E-06 | 1.37E-05 | 3.343685 |
| SERPING1 | 2.487515 | 5.074993 | 4.126571 | 4.28E-05 | 0.000109 | 1.224159 |
| RNASE3 | 2.457313 | 5.563634 | 5.486981 | 6.35E-08 | 2.46E-07 | 7.454061 |
| CST7 | 2.453037 | 5.736082 | 10.04976 | 7.31E-22 | 2.37E-20 | 38.85637 |
| SAMSN1 | 2.395851 | 5.407945 | 8.645136 | 6.43E-17 | 9.31E-16 | 27.66026 |
| FKBP5 | 2.394175 | 5.386197 | 8.835994 | 1.47E-17 | 2.40E-16 | 29.10974 |
| C3AR1 | 2.389854 | 5.752038 | 8.626868 | 7.40E-17 | 1.06E-15 | 27.52276 |
| DAAM2 | 2.373 | 4.813908 | 7.045505 | 5.79E-12 | 3.88E-11 | 16.49 |
| S100P | 2.357281 | 6.490492 | 8.897426 | 9.10E-18 | 1.54E-16 | 29.58128 |
| NAIP | 2.337347 | 5.633032 | 7.735987 | 5.23E-14 | 4.79E-13 | 21.09011 |
| SLPI | 2.33535 | 5.45006 | 5.664447 | 2.43E-08 | 9.94E-08 | 8.383944 |
| FAM20A | 2.311868 | 5.290991 | 5.425315 | 8.82E-08 | 3.33E-07 | 7.137115 |
| PLIN3 | 2.262734 | 5.84634 | 7.120855 | 3.52E-12 | 2.43E-11 | 16.97492 |
| PYGL | 2.261332 | 5.835206 | 10.09373 | 5.02E-22 | 1.70E-20 | 39.2258 |
| CTSG | 2.207514 | 5.439478 | 2.577862 | 0.010211 | 0.017544 | -3.82992 |
| PROK2 | 2.203958 | 5.663994 | 9.457749 | 1.02E-19 | 2.36E-18 | 33.99152 |
| B4GALT5 | 2.195363 | 5.571241 | 10.88094 | 5.10E-25 | 3.05E-23 | 46.01779 |
| LILRA5 | 2.188836 | 5.366748 | 8.198029 | 1.86E-15 | 2.14E-14 | 24.35869 |
| RSAD2 | 2.170266 | 5.328793 | 3.531522 | 0.000449 | 0.000979 | -0.98193 |
| ACSL1 | 2.131728 | 5.824304 | 11.32691 | 8.90E-27 | 7.08E-25 | 50.00847 |
| IL18RAP | 2.115962 | 5.768333 | 8.806459 | 1.85E-17 | 2.92E-16 | 28.88389 |
| FCAR | 2.084855 | 4.929137 | 9.424522 | 1.34E-19 | 3.03E-18 | 33.72461 |
| PFKFB2 | 2.060825 | 4.65433 | 7.579826 | 1.56E-13 | 1.33E-12 | 20.01938 |
| SERPINA1 | 2.017298 | 4.991029 | 8.811027 | 1.79E-17 | 2.84E-16 | 28.91879 |
| CKAP4 | 2.016307 | 4.736007 | 9.124739 | 1.51E-18 | 2.95E-17 | 31.34692 |
| PCOLCE2 | 2.000051 | 5.076758 | 2.612327 | 0.009249 | 0.016029 | -3.74202 |
| SLC22A4 | 1.985148 | 5.435402 | 10.60366 | 6.00E-24 | 2.96E-22 | 43.58766 |
| MAPK14 | 1.969996 | 5.22706 | 9.398469 | 1.66E-19 | 3.71E-18 | 33.5158 |
| RNASE2 | 1.964111 | 5.023245 | 7.881953 | 1.85E-14 | 1.83E-13 | 22.1066 |
| OSCAR | 1.959758 | 5.098916 | 10.35043 | 5.50E-23 | 2.31E-21 | 41.40382 |
| BCL6 | 1.93699 | 5.369266 | 10.27767 | 1.03E-22 | 4.11E-21 | 40.78274 |
| GRB10 | 1.936904 | 4.527747 | 8.583129 | 1.03E-16 | 1.43E-15 | 27.19444 |
| SERPINB1 | 1.908474 | 6.085235 | 9.816794 | 5.24E-21 | 1.48E-19 | 36.91764 |
| SMPDL3A | 1.884972 | 4.581748 | 7.017294 | 6.96E-12 | 4.61E-11 | 16.30955 |
| MMRN1 | 1.881864 | 4.200731 | 4.710836 | 3.16E-06 | 9.64E-06 | 3.701525 |
| PLSCR1 | 1.878816 | 4.96429 | 9.524689 | 5.92E-20 | 1.41E-18 | 34.53126 |
| FPR2 | 1.869019 | 4.956422 | 9.24903 | 5.57E-19 | 1.17E-17 | 32.32602 |
| XK | 1.861467 | 5.270619 | 4.760112 | 2.50E-06 | 7.74E-06 | 3.924285 |
| GYPA | 1.858015 | 5.213846 | 4.024968 | 6.53E-05 | 0.000162 | 0.824586 |
| SERPINB2 | 1.854327 | 4.690721 | 5.713659 | 1.85E-08 | 7.70E-08 | 8.646448 |
| ST6GALNAC3 | 1.851759 | 4.630573 | 7.751971 | 4.67E-14 | 4.32E-13 | 21.20068 |
| DACH1 | 1.849798 | 5.307308 | 7.166472 | 2.60E-12 | 1.83E-11 | 17.27055 |
| SIGLEC5 | 1.831525 | 4.899769 | 10.8576 | 6.28E-25 | 3.64E-23 | 45.81164 |
| CLIC2 | 1.827047 | 4.571456 | 4.282012 | 2.20E-05 | 5.91E-05 | 1.85349 |
| VSIG4 | 1.808387 | 4.974799 | 3.703957 | 0.000235 | 0.000534 | -0.37607 |
| FBXO6 | 1.804705 | 4.528301 | 5.845659 | 8.84E-09 | 3.89E-08 | 9.360432 |
| RHAG | 1.801125 | 3.852442 | 6.504115 | 1.81E-10 | 1.01E-09 | 13.13296 |
| LTB4R | 1.787891 | 5.051309 | 10.22016 | 1.70E-22 | 6.50E-21 | 40.29392 |
| FFAR2 | 1.783494 | 4.855709 | 7.967306 | 1.00E-14 | 1.03E-13 | 22.70793 |
| UPP1 | 1.772617 | 5.080544 | 9.483063 | 8.33E-20 | 1.95E-18 | 34.19531 |
| CDA | 1.76281 | 5.25131 | 9.50173 | 7.15E-20 | 1.68E-18 | 34.34584 |
| SLC25A37 | 1.762579 | 4.478122 | 8.005138 | 7.63E-15 | 8.01E-14 | 22.97608 |
| F5 | 1.761153 | 4.257678 | 8.178835 | 2.14E-15 | 2.43E-14 | 24.21996 |
| MANSC1 | 1.727178 | 4.284779 | 8.048169 | 5.58E-15 | 5.97E-14 | 23.2823 |
| FLOT2 | 1.717804 | 4.885082 | 7.008335 | 7.38E-12 | 4.86E-11 | 16.25237 |
| TMEM158 | 1.666489 | 4.631488 | 4.251761 | 2.51E-05 | 6.67E-05 | 1.72931 |
| PADI4 | 1.664344 | 5.709044 | 8.31269 | 7.95E-16 | 9.64E-15 | 25.1926 |
| STOM | 1.662052 | 4.732159 | 11.06269 | 9.91E-26 | 6.42E-24 | 47.63215 |
| BST1 | 1.651384 | 5.198562 | 9.079389 | 2.16E-18 | 4.10E-17 | 30.99207 |
| HIST2H2AA3 | 1.647831 | 5.169508 | 10.50271 | 1.46E-23 | 6.91E-22 | 42.71289 |
| CEBPD | 1.607796 | 4.837087 | 8.386285 | 4.59E-16 | 5.67E-15 | 25.73253 |
| SOCS3 | 1.606609 | 3.906845 | 9.824929 | 4.89E-21 | 1.39E-19 | 36.98481 |
| SIPA1L2 | 1.586479 | 4.184532 | 9.647981 | 2.14E-20 | 5.47E-19 | 35.53238 |
| G0S2 | 1.584495 | 4.156764 | 5.72293 | 1.76E-08 | 7.35E-08 | 8.696126 |
| FCER1G | 1.582112 | 5.995805 | 9.215291 | 7.31E-19 | 1.49E-17 | 32.05929 |
| GAS7 | 1.574699 | 4.666584 | 9.693398 | 1.47E-20 | 3.86E-19 | 35.90343 |
| LIN7A | 1.573043 | 4.959591 | 8.184472 | 2.06E-15 | 2.34E-14 | 24.26068 |
| MKNK1 | 1.567986 | 4.619867 | 10.06235 | 6.56E-22 | 2.16E-20 | 38.96204 |
| BPGM | 1.566591 | 5.93358 | 3.721365 | 0.000219 | 0.000502 | -0.31338 |
| TXN | 1.564586 | 4.545621 | 8.024532 | 6.63E-15 | 7.04E-14 | 23.11394 |
| LILRA3 | 1.563521 | 4.59482 | 6.562839 | 1.26E-10 | 7.14E-10 | 13.48616 |
| KIF1B | 1.560094 | 4.200444 | 11.56655 | 9.72E-28 | 9.41E-26 | 52.19298 |
| CLEC1B | 1.548429 | 4.224028 | 6.078411 | 2.33E-09 | 1.12E-08 | 10.65412 |
| HSPA1A | 1.535279 | 5.485369 | 8.705349 | 4.05E-17 | 6.09E-16 | 28.11499 |
| HGF | 1.528528 | 4.418887 | 4.195241 | 3.20E-05 | 8.34E-05 | 1.499498 |
| ITGAM | 1.516853 | 5.340426 | 10.20305 | 1.97E-22 | 7.48E-21 | 40.14884 |
| ROPN1L | 1.489202 | 3.499499 | 8.810116 | 1.80E-17 | 2.85E-16 | 28.91183 |
| DGAT2 | 1.485575 | 4.738088 | 10.22776 | 1.59E-22 | 6.14E-21 | 40.35844 |
| GYPB | 1.480817 | 5.199668 | 4.46601 | 9.75E-06 | 2.78E-05 | 2.626441 |
| AIM2 | 1.479219 | 4.118527 | 6.046308 | 2.80E-09 | 1.34E-08 | 10.47307 |
| CR1 | 1.473281 | 4.139391 | 10.69804 | 2.60E-24 | 1.36E-22 | 44.4103 |
| THBS1 | 1.46752 | 3.856278 | 5.756495 | 1.46E-08 | 6.17E-08 | 8.876573 |
| PGS1 | 1.45933 | 5.012144 | 9.344924 | 2.57E-19 | 5.63E-18 | 33.08792 |
| C1QC | 1.456633 | 3.513247 | 4.423958 | 1.18E-05 | 3.30E-05 | 2.447124 |
| CA1 | 1.452823 | 5.153253 | 5.391526 | 1.05E-07 | 3.93E-07 | 6.964804 |
| NSUN7 | 1.448273 | 3.864481 | 9.248546 | 5.59E-19 | 1.17E-17 | 32.32218 |
| CLEC4E | 1.446457 | 4.656468 | 8.697677 | 4.30E-17 | 6.41E-16 | 28.05692 |
| LRRN1 | 1.443927 | 4.056133 | 5.625428 | 3.00E-08 | 1.22E-07 | 8.177242 |
| CPEB4 | 1.428991 | 4.532834 | 9.858806 | 3.68E-21 | 1.08E-19 | 37.26497 |
| C1QA | 1.425666 | 4.499887 | 3.437649 | 0.000633 | 0.001352 | -1.30018 |
| S100A9 | 1.425166 | 6.118953 | 10.09414 | 5.00E-22 | 1.70E-20 | 39.22927 |
| LIMK2 | 1.41962 | 4.29678 | 9.290924 | 3.98E-19 | 8.48E-18 | 32.65818 |
| ECHDC3 | 1.415455 | 3.89222 | 6.581476 | 1.12E-10 | 6.42E-10 | 13.59881 |
| ATP8B4 | 1.413313 | 4.709431 | 5.402626 | 9.95E-08 | 3.72E-07 | 7.021306 |
| NFIL3 | 1.403478 | 3.95221 | 9.51719 | 6.30E-20 | 1.50E-18 | 34.47066 |
| PIK3AP1 | 1.395408 | 3.761784 | 7.750216 | 4.73E-14 | 4.36E-13 | 21.18853 |
| KCNJ15 | 1.387287 | 4.292971 | 9.444427 | 1.14E-19 | 2.62E-18 | 33.88442 |
| WDFY3 | 1.383209 | 4.379254 | 11.50825 | 1.67E-27 | 1.54E-25 | 51.65907 |
| MCTP2 | 1.382771 | 4.265526 | 10.11423 | 4.21E-22 | 1.47E-20 | 39.39835 |
| HPSE | 1.38079 | 4.037713 | 7.048273 | 5.68E-12 | 3.82E-11 | 16.50774 |
| BCAT1 | 1.366535 | 4.013417 | 7.37973 | 6.19E-13 | 4.84E-12 | 18.67311 |
| CA2 | 1.363729 | 4.089567 | 4.278707 | 2.23E-05 | 5.99E-05 | 1.839885 |
| PI3 | 1.361959 | 4.970705 | 2.772708 | 0.005755 | 0.010375 | -3.31803 |
| TLR2 | 1.360511 | 4.842432 | 8.996383 | 4.18E-18 | 7.51E-17 | 30.34592 |
| ERLIN1 | 1.35903 | 3.988146 | 7.580305 | 1.56E-13 | 1.32E-12 | 20.02264 |
| LMO2 | 1.352395 | 4.852921 | 6.791827 | 2.99E-11 | 1.83E-10 | 14.88899 |
| TLR8 | 1.34944 | 4.654022 | 9.556714 | 4.55E-20 | 1.10E-18 | 34.79043 |
| SPI1 | 1.342984 | 4.382748 | 8.945664 | 6.23E-18 | 1.08E-16 | 29.95323 |
| IL1RN | 1.342818 | 4.337214 | 7.996174 | 8.14E-15 | 8.52E-14 | 22.91246 |
| CD63 | 1.342755 | 5.095298 | 9.201545 | 8.16E-19 | 1.64E-17 | 31.95083 |
| IFITM3 | 1.342116 | 4.648294 | 10.21295 | 1.81E-22 | 6.89E-21 | 40.23281 |
| RAB13 | 1.340408 | 4.510502 | 6.816134 | 2.56E-11 | 1.58E-10 | 15.04027 |
| NQO2 | 1.339437 | 3.874233 | 9.135803 | 1.38E-18 | 2.72E-17 | 31.43369 |
| SEMA4A | 1.335133 | 4.359213 | 9.775435 | 7.40E-21 | 2.03E-19 | 36.57671 |
| HCK | 1.330112 | 4.770926 | 9.081507 | 2.13E-18 | 4.05E-17 | 31.00861 |
| QPCT | 1.329565 | 4.671641 | 8.691338 | 4.51E-17 | 6.69E-16 | 28.00897 |
| WIPI1 | 1.321082 | 4.204581 | 9.876228 | 3.18E-21 | 9.44E-20 | 37.4093 |
| GK | 1.318261 | 4.057292 | 7.945184 | 1.18E-14 | 1.19E-13 | 22.55158 |
| IFI44L | 1.317621 | 4.442261 | 2.881296 | 0.004121 | 0.007618 | -3.01703 |
| TSPO | 1.316056 | 5.412053 | 8.586524 | 1.01E-16 | 1.39E-15 | 27.21988 |
| OPLAH | 1.31539 | 3.576665 | 8.93353 | 6.85E-18 | 1.17E-16 | 29.85953 |
| NCF4 | 1.291755 | 4.977862 | 9.999842 | 1.12E-21 | 3.57E-20 | 38.43838 |
| TIMP1 | 1.284524 | 5.168673 | 7.682911 | 7.61E-14 | 6.77E-13 | 20.72423 |
| STXBP2 | 1.280399 | 3.871102 | 8.593214 | 9.57E-17 | 1.33E-15 | 27.27003 |
| CD55 | 1.276384 | 5.107937 | 9.564285 | 4.27E-20 | 1.05E-18 | 34.85179 |
| CEP55 | 1.275633 | 4.185091 | 4.686988 | 3.53E-06 | 1.07E-05 | 3.59448 |
| SIRPA | 1.275336 | 4.727445 | 10.62192 | 5.10E-24 | 2.55E-22 | 43.74644 |
| FLOT1 | 1.257983 | 4.903956 | 9.219657 | 7.06E-19 | 1.44E-17 | 32.09377 |
| SUCNR1 | 1.248294 | 4.086716 | 2.984939 | 0.002968 | 0.005631 | -2.71931 |
| GALNT14 | 1.247992 | 3.81242 | 6.380486 | 3.86E-10 | 2.06E-09 | 12.39826 |
| CPD | 1.241697 | 3.935158 | 9.081283 | 2.13E-18 | 4.05E-17 | 31.00687 |
| SIGLEC9 | 1.232783 | 3.979873 | 9.544335 | 5.04E-20 | 1.21E-18 | 34.69018 |
| KLHL2 | 1.22964 | 3.777576 | 7.340814 | 8.07E-13 | 6.18E-12 | 18.41466 |
| AQP9 | 1.227934 | 5.093886 | 10.29353 | 9.01E-23 | 3.66E-21 | 40.91786 |
| RAB31 | 1.227193 | 4.836748 | 9.433948 | 1.24E-19 | 2.83E-18 | 33.80026 |
| WSB1 | 1.223621 | 3.875393 | 8.595031 | 9.44E-17 | 1.31E-15 | 27.28365 |
| METTL9 | 1.222846 | 5.096487 | 11.40712 | 4.26E-27 | 3.60E-25 | 50.73657 |
| FPR1 | 1.222202 | 4.786517 | 8.529103 | 1.56E-16 | 2.09E-15 | 26.79064 |
| HBZ | 1.217895 | 4.500727 | 2.909422 | 0.003773 | 0.007038 | -2.93724 |
| DSC2 | 1.209628 | 4.246439 | 6.499237 | 1.87E-10 | 1.03E-09 | 13.10375 |
| GCA | 1.196563 | 5.064899 | 9.741624 | 9.82E-21 | 2.67E-19 | 36.29875 |
| SERPINB10 | 1.195837 | 3.756672 | 5.288051 | 1.81E-07 | 6.54E-07 | 6.443138 |
| PRKCD | 1.193816 | 4.592339 | 8.413096 | 3.75E-16 | 4.68E-15 | 25.93013 |
| FES | 1.190116 | 4.200496 | 8.993985 | 4.26E-18 | 7.64E-17 | 30.32731 |
| GRN | 1.187517 | 4.804478 | 7.338638 | 8.19E-13 | 6.26E-12 | 18.40024 |
| KREMEN1 | 1.187377 | 4.096349 | 8.633295 | 7.05E-17 | 1.01E-15 | 27.57111 |
| TIMP2 | 1.184967 | 4.639076 | 8.581429 | 1.05E-16 | 1.44E-15 | 27.18171 |
| AGTRAP | 1.183611 | 4.525155 | 9.051195 | 2.71E-18 | 5.05E-17 | 30.77211 |
| HIST1H1C | 1.18261 | 4.004062 | 6.368934 | 4.14E-10 | 2.20E-09 | 12.33022 |
| ALOX5AP | 1.181034 | 5.342869 | 10.83291 | 7.83E-25 | 4.46E-23 | 45.59399 |
| GCLM | 1.180488 | 3.927411 | 9.293566 | 3.89E-19 | 8.32E-18 | 32.67916 |
| IL1R1 | 1.180076 | 3.471874 | 5.6368 | 2.82E-08 | 1.15E-07 | 8.237358 |
| PGD | 1.175596 | 4.419232 | 10.31338 | 7.59E-23 | 3.15E-21 | 41.08721 |
| CTSD | 1.170674 | 4.414811 | 8.055121 | 5.31E-15 | 5.70E-14 | 23.33189 |
| CD274 | 1.170565 | 3.48819 | 4.499061 | 8.40E-06 | 2.42E-05 | 2.768478 |
| GBA | 1.168331 | 3.728345 | 8.891475 | 9.53E-18 | 1.60E-16 | 29.53549 |
| S100A8 | 1.155294 | 5.297879 | 7.877706 | 1.91E-14 | 1.88E-13 | 22.07681 |
| ZYX | 1.150915 | 4.142737 | 7.277224 | 1.24E-12 | 9.26E-12 | 17.99475 |
| HIST1H2BD | 1.150166 | 4.26334 | 9.116036 | 1.62E-18 | 3.13E-17 | 31.27872 |
| CARD6 | 1.139704 | 4.11298 | 8.106563 | 3.65E-15 | 4.03E-14 | 23.69989 |
| BASP1 | 1.13786 | 5.040141 | 10.37921 | 4.28E-23 | 1.84E-21 | 41.65024 |
| G6PD | 1.135058 | 4.001904 | 8.606262 | 8.67E-17 | 1.21E-15 | 27.36793 |
| SULT1B1 | 1.134699 | 4.103463 | 6.941572 | 1.14E-11 | 7.36E-11 | 15.82815 |
| CCR1 | 1.134091 | 4.334064 | 8.529195 | 1.56E-16 | 2.09E-15 | 26.79133 |
| PROS1 | 1.131404 | 3.438057 | 5.915254 | 5.96E-09 | 2.70E-08 | 9.742629 |
| PPP1R3D | 1.130086 | 3.676105 | 9.867098 | 3.43E-21 | 1.01E-19 | 37.33364 |
| ASPH | 1.128203 | 4.011595 | 6.296065 | 6.42E-10 | 3.33E-09 | 11.90349 |
| GNS | 1.1251 | 4.41753 | 7.813001 | 3.03E-14 | 2.90E-13 | 21.62455 |
| ZNF438 | 1.120127 | 3.833553 | 9.095594 | 1.90E-18 | 3.65E-17 | 31.11872 |
| MARCKS | 1.118557 | 4.041938 | 8.643713 | 6.50E-17 | 9.39E-16 | 27.64954 |
| FGD4 | 1.116095 | 3.792484 | 7.405333 | 5.20E-13 | 4.13E-12 | 18.84374 |
| CD14 | 1.1026 | 4.746047 | 6.113768 | 1.89E-09 | 9.25E-09 | 10.85449 |
| HIP1 | 1.094942 | 3.711699 | 8.279426 | 1.02E-15 | 1.21E-14 | 24.94976 |
| ALPK1 | 1.092518 | 3.923841 | 9.720687 | 1.17E-20 | 3.14E-19 | 36.12695 |
| CASP5 | 1.090544 | 3.802203 | 6.871391 | 1.80E-11 | 1.13E-10 | 15.38586 |
| RALB | 1.089051 | 4.115803 | 8.704798 | 4.07E-17 | 6.11E-16 | 28.11082 |
| TMCC2 | 1.086812 | 3.79165 | 5.182806 | 3.12E-07 | 1.09E-06 | 5.921875 |
| TLR4 | 1.086743 | 4.104418 | 7.715432 | 6.05E-14 | 5.48E-13 | 20.94817 |
| P2RX1 | 1.085649 | 4.150381 | 8.883131 | 1.02E-17 | 1.71E-16 | 29.47133 |
| C5AR1 | 1.08504 | 3.82735 | 9.296945 | 3.79E-19 | 8.12E-18 | 32.70601 |
| CREB5 | 1.081747 | 4.248135 | 7.671337 | 8.25E-14 | 7.29E-13 | 20.64471 |
| RRM2 | 1.075175 | 4.109429 | 3.800351 | 0.000161 | 0.000377 | -0.02544 |
| CSF3R | 1.069567 | 4.737709 | 7.247977 | 1.51E-12 | 1.11E-11 | 17.80263 |
| IGSF6 | 1.068402 | 4.689196 | 7.759068 | 4.45E-14 | 4.13E-13 | 21.24983 |
| RRAGD | 1.067645 | 4.11776 | 8.578736 | 1.07E-16 | 1.46E-15 | 27.16154 |
| LY96 | 1.062903 | 4.71177 | 6.211689 | 1.06E-09 | 5.36E-09 | 11.41466 |
| MTF1 | 1.06206 | 4.683331 | 10.38016 | 4.25E-23 | 1.84E-21 | 41.65838 |
| IL1RAP | 1.06161 | 3.241782 | 6.915304 | 1.35E-11 | 8.64E-11 | 15.66217 |
| FAM126B | 1.059606 | 4.080389 | 7.006789 | 7.46E-12 | 4.90E-11 | 16.2425 |
| LMNB1 | 1.05934 | 3.88569 | 8.581219 | 1.05E-16 | 1.44E-15 | 27.18013 |
| ADAM9 | 1.055391 | 3.720759 | 8.249386 | 1.27E-15 | 1.50E-14 | 24.7311 |
| POR | 1.055313 | 3.28538 | 9.833004 | 4.57E-21 | 1.30E-19 | 37.05154 |
| TPST1 | 1.05494 | 3.631691 | 5.679546 | 2.23E-08 | 9.19E-08 | 8.464275 |
| JUNB | 1.053656 | 4.130885 | 9.907022 | 2.45E-21 | 7.40E-20 | 37.66484 |
| NFE2 | 1.053516 | 4.589512 | 9.467549 | 9.46E-20 | 2.19E-18 | 34.07037 |
| ETS2 | 1.047737 | 3.870409 | 9.071265 | 2.31E-18 | 4.34E-17 | 30.92864 |
| ENTPD7 | 1.047332 | 3.537181 | 7.14756 | 2.95E-12 | 2.06E-11 | 17.1478 |
| SPTLC2 | 1.046976 | 3.826035 | 8.090695 | 4.09E-15 | 4.49E-14 | 23.58618 |
| BATF2 | 1.046142 | 3.865027 | 5.080028 | 5.24E-07 | 1.79E-06 | 5.421968 |
| DOK3 | 1.035057 | 3.624636 | 11.07748 | 8.67E-26 | 5.69E-24 | 47.76428 |
| VSTM1 | 1.034016 | 4.215576 | 5.305792 | 1.65E-07 | 5.99E-07 | 6.531934 |
| CTSA | 1.031046 | 4.386681 | 7.816413 | 2.96E-14 | 2.83E-13 | 21.64832 |
| CHPT1 | 1.030434 | 4.579576 | 7.175371 | 2.45E-12 | 1.74E-11 | 17.32841 |
| TBXAS1 | 1.030333 | 4.221973 | 7.463224 | 3.50E-13 | 2.86E-12 | 19.23133 |
| FGR | 1.026954 | 4.67954 | 8.964661 | 5.37E-18 | 9.42E-17 | 30.10013 |
| GPR160 | 1.022635 | 4.197299 | 8.555257 | 1.28E-16 | 1.73E-15 | 26.98589 |
| HIST2H2BE | 1.019489 | 4.261804 | 7.069147 | 4.96E-12 | 3.36E-11 | 16.64169 |
| PLXNC1 | 1.019484 | 3.876306 | 8.922557 | 7.47E-18 | 1.27E-16 | 29.77488 |
| ALOX5 | 1.016663 | 4.858827 | 8.979058 | 4.79E-18 | 8.48E-17 | 30.2116 |
| GLT1D1 | 1.015416 | 4.858227 | 8.264893 | 1.14E-15 | 1.35E-14 | 24.8439 |
| LPCAT2 | 1.012799 | 3.999907 | 8.799772 | 1.95E-17 | 3.07E-16 | 28.83284 |
| ATP6V1C1 | 1.012132 | 3.827432 | 8.526481 | 1.59E-16 | 2.12E-15 | 26.7711 |
| B3GNT5 | 1.010975 | 3.304978 | 7.483946 | 3.03E-13 | 2.50E-12 | 19.37066 |
| TBC1D8 | 1.008343 | 4.004532 | 9.034278 | 3.10E-18 | 5.72E-17 | 30.64037 |
| TSPAN2 | 1.006323 | 3.994855 | 5.779594 | 1.28E-08 | 5.50E-08 | 9.001298 |
| DENND3 | 1.001864 | 4.136856 | 9.367813 | 2.13E-19 | 4.74E-18 | 33.27061 |
| CCNA1 | 1.001207 | 3.89251 | 2.944231 | 0.00338 | 0.006358 | -2.83746 |
| IMPA2 | 1.000173 | 4.424291 | 7.684127 | 7.54E-14 | 6.72E-13 | 20.73259 |
| TP53I3 | 0.999517 | 3.673896 | 7.045121 | 5.80E-12 | 3.89E-11 | 16.48754 |
| NAMPT | 0.998442 | 4.324914 | 6.880141 | 1.70E-11 | 1.08E-10 | 15.4408 |
| HPGD | 0.998265 | 4.80697 | 2.931669 | 0.003517 | 0.006596 | -2.8736 |
| RNF24 | 0.992043 | 4.259601 | 9.853864 | 3.84E-21 | 1.11E-19 | 37.22406 |
| BAZ1A | 0.990933 | 4.057421 | 8.703044 | 4.12E-17 | 6.18E-16 | 28.09754 |
| IL1B | 0.986018 | 4.300227 | 8.418279 | 3.61E-16 | 4.52E-15 | 25.96839 |
| TMEM120A | 0.981821 | 4.076068 | 9.044218 | 2.86E-18 | 5.31E-17 | 30.71776 |
| PRC1 | 0.980146 | 3.424019 | 5.360106 | 1.24E-07 | 4.59E-07 | 6.805443 |
| HIST1H4H | 0.975967 | 3.481519 | 7.025061 | 6.62E-12 | 4.39E-11 | 16.35917 |
| RAB27A | 0.974266 | 4.586156 | 8.793143 | 2.05E-17 | 3.22E-16 | 28.78225 |
| SH3GLB1 | 0.973037 | 4.203137 | 9.270644 | 4.68E-19 | 9.90E-18 | 32.49725 |
| CCL3 | 0.972174 | 3.286857 | 2.468888 | 0.013869 | 0.023164 | -4.10037 |
| CNIH4 | 0.971711 | 4.454069 | 9.40419 | 1.59E-19 | 3.55E-18 | 33.56161 |
| EXOSC4 | 0.969504 | 3.802184 | 7.81086 | 3.08E-14 | 2.93E-13 | 21.60964 |
| FUT7 | 0.968467 | 3.068603 | 5.994345 | 3.79E-09 | 1.77E-08 | 10.18178 |
| MAN1A1 | 0.966805 | 4.049541 | 7.049336 | 5.64E-12 | 3.80E-11 | 16.51455 |
| ABHD2 | 0.96566 | 3.644975 | 7.221042 | 1.81E-12 | 1.31E-11 | 17.62624 |
| S100A11 | 0.963784 | 4.771642 | 8.895979 | 9.20E-18 | 1.55E-16 | 29.57014 |
| CCPG1 | 0.959087 | 4.379797 | 7.437139 | 4.18E-13 | 3.38E-12 | 19.05638 |
| OASL | 0.951874 | 3.683786 | 2.872484 | 0.004236 | 0.007818 | -3.04188 |
| DNAJC5 | 0.951005 | 2.830488 | 8.004774 | 7.65E-15 | 8.02E-14 | 22.9735 |
| TGFB1I1 | 0.950257 | 3.413174 | 4.433757 | 1.13E-05 | 3.18E-05 | 2.488767 |
| RHCE | 0.948654 | 4.071812 | 4.928569 | 1.11E-06 | 3.64E-06 | 4.701847 |
| STX11 | 0.947519 | 4.600241 | 7.237619 | 1.62E-12 | 1.18E-11 | 17.73473 |
| SLC40A1 | 0.942846 | 4.212553 | 10.51385 | 1.32E-23 | 6.33E-22 | 42.8092 |
| RAB32 | 0.935876 | 4.209527 | 8.452897 | 2.78E-16 | 3.59E-15 | 26.22436 |
| SIAH2 | 0.933232 | 4.574744 | 5.938336 | 5.22E-09 | 2.39E-08 | 9.87027 |
| TSHZ3 | 0.932249 | 3.84277 | 7.76986 | 4.12E-14 | 3.86E-13 | 21.32465 |
| GGH | 0.931711 | 3.504409 | 5.451968 | 7.66E-08 | 2.92E-07 | 7.27371 |
| KCNJ2 | 0.931444 | 3.685708 | 8.049821 | 5.52E-15 | 5.90E-14 | 23.29408 |
| CHSY1 | 0.930859 | 3.882828 | 7.750632 | 4.72E-14 | 4.35E-13 | 21.19141 |
| CTNNAL1 | 0.925727 | 3.616894 | 4.252775 | 2.50E-05 | 6.65E-05 | 1.73346 |
| PDGFC | 0.924391 | 3.426081 | 5.401128 | 1.00E-07 | 3.75E-07 | 7.013672 |
| HLX | 0.924234 | 4.100313 | 9.123666 | 1.52E-18 | 2.96E-17 | 31.33851 |
| NLRP12 | 0.923095 | 4.342531 | 8.53182 | 1.53E-16 | 2.05E-15 | 26.81091 |
| STX3 | 0.922323 | 3.515996 | 7.146656 | 2.97E-12 | 2.08E-11 | 17.14194 |
| SLC7A5 | 0.918179 | 3.510943 | 5.821513 | 1.01E-08 | 4.42E-08 | 9.228756 |
| CD58 | 0.909409 | 4.171336 | 8.283366 | 9.89E-16 | 1.18E-14 | 24.97848 |
| H1F0 | 0.907953 | 3.449488 | 7.636276 | 1.05E-13 | 9.20E-13 | 20.40441 |
| GNA15 | 0.906521 | 3.32456 | 9.235482 | 6.21E-19 | 1.29E-17 | 32.21882 |
| ITGA7 | 0.90599 | 3.558339 | 4.26247 | 2.39E-05 | 6.39E-05 | 1.773176 |
| CEBPB | 0.905166 | 4.801701 | 8.058956 | 5.16E-15 | 5.57E-14 | 23.35926 |
| SHKBP1 | 0.904226 | 3.842087 | 7.390122 | 5.77E-13 | 4.55E-12 | 18.74231 |
| VNN2 | 0.901882 | 4.783589 | 7.039042 | 6.04E-12 | 4.02E-11 | 16.44861 |
| LAMP3 | 0.901578 | 3.139801 | 2.494091 | 0.012932 | 0.021754 | -4.03883 |
| DPY19L3 | 0.896447 | 3.126735 | 5.463217 | 7.21E-08 | 2.77E-07 | 7.331542 |
| CD24 | 0.896036 | 4.132737 | 4.976807 | 8.76E-07 | 2.91E-06 | 4.929047 |
| CRISPLD2 | 0.895517 | 3.546903 | 8.8273 | 1.57E-17 | 2.55E-16 | 29.0432 |
| SLC22A15 | 0.88843 | 3.646406 | 8.619835 | 7.81E-17 | 1.10E-15 | 27.46988 |
| GAPDH | 0.8874 | 4.779063 | 7.305256 | 1.03E-12 | 7.74E-12 | 18.17949 |
| IDI1 | 0.885204 | 4.203731 | 7.390048 | 5.77E-13 | 4.55E-12 | 18.74181 |
| RAB20 | 0.88324 | 3.426987 | 9.567522 | 4.16E-20 | 1.02E-18 | 34.87804 |
| DDAH2 | 0.880353 | 3.27836 | 8.848028 | 1.34E-17 | 2.22E-16 | 29.20192 |
| UHRF1 | 0.880235 | 3.45624 | 3.88436 | 0.000116 | 0.000276 | 0.287086 |
| IL17RA | 0.876021 | 4.240269 | 9.97205 | 1.41E-21 | 4.46E-20 | 38.20625 |
| PLP2 | 0.868629 | 4.578722 | 7.989272 | 8.56E-15 | 8.93E-14 | 22.8635 |
| CAPN3 | 0.867345 | 3.481371 | 8.343917 | 6.30E-16 | 7.69E-15 | 25.42126 |
| ARRB2 | 0.865896 | 3.719389 | 8.235575 | 1.41E-15 | 1.65E-14 | 24.63077 |
| MPP1 | 0.861806 | 4.582197 | 6.146519 | 1.56E-09 | 7.73E-09 | 11.04098 |
| NUMB | 0.86116 | 4.338287 | 7.486003 | 2.99E-13 | 2.46E-12 | 19.3845 |
| ACSL4 | 0.859898 | 3.787408 | 7.764822 | 4.27E-14 | 3.98E-13 | 21.28971 |
| ANKRD13A | 0.859874 | 3.772957 | 4.821995 | 1.86E-06 | 5.87E-06 | 4.207051 |
| CEACAM4 | 0.857311 | 3.443989 | 9.572944 | 3.98E-20 | 9.80E-19 | 34.92201 |
| SIGLEC10 | 0.854991 | 3.962944 | 4.882587 | 1.39E-06 | 4.49E-06 | 4.487155 |
| MXD1 | 0.854274 | 3.74257 | 9.426622 | 1.32E-19 | 2.99E-18 | 33.74146 |
| MTX1 | 0.852687 | 3.89129 | 8.485472 | 2.17E-16 | 2.86E-15 | 26.46596 |
| NARF | 0.851219 | 3.906715 | 8.103812 | 3.72E-15 | 4.11E-14 | 23.68016 |
| IER3 | 0.851126 | 3.650918 | 7.554749 | 1.86E-13 | 1.57E-12 | 19.84906 |
| CDC42EP3 | 0.850721 | 3.696685 | 7.686729 | 7.40E-14 | 6.61E-13 | 20.75049 |
| FFAR3 | 0.850565 | 3.559701 | 5.399066 | 1.01E-07 | 3.79E-07 | 7.003174 |
| SLA | 0.849822 | 4.521357 | 7.132521 | 3.26E-12 | 2.26E-11 | 17.05037 |
| BSG | 0.849234 | 4.665257 | 5.356248 | 1.27E-07 | 4.67E-07 | 6.785935 |
| MAP2K6 | 0.8492 | 4.000193 | 5.744276 | 1.56E-08 | 6.59E-08 | 8.810776 |
| CKLF | 0.84913 | 4.717201 | 6.437433 | 2.73E-10 | 1.48E-09 | 12.73519 |
| SRPK1 | 0.847003 | 4.040141 | 10.30385 | 8.24E-23 | 3.38E-21 | 41.00592 |
| TCTEX1D1 | 0.843709 | 3.135998 | 5.469554 | 6.97E-08 | 2.68E-07 | 7.364163 |
| RIT1 | 0.843007 | 3.492209 | 7.177625 | 2.42E-12 | 1.72E-11 | 17.34307 |
| TCIRG1 | 0.842678 | 4.220779 | 8.663513 | 5.59E-17 | 8.17E-16 | 27.79879 |
| PRAM1 | 0.841767 | 4.363413 | 6.651914 | 7.24E-11 | 4.24E-10 | 14.02703 |
| LYN | 0.837744 | 4.278274 | 7.709598 | 6.30E-14 | 5.69E-13 | 20.90795 |
| LILRB3 | 0.835098 | 4.312579 | 8.092927 | 4.03E-15 | 4.44E-14 | 23.60216 |
| HMMR | 0.831696 | 3.871014 | 4.737674 | 2.78E-06 | 8.56E-06 | 3.822589 |
| APOBEC3A | 0.831173 | 3.69331 | 6.829325 | 2.35E-11 | 1.46E-10 | 15.12256 |
| BCL3 | 0.828116 | 4.091576 | 10.99797 | 1.78E-25 | 1.08E-23 | 47.05535 |
| CD59 | 0.827193 | 3.947559 | 7.234414 | 1.65E-12 | 1.20E-11 | 17.71374 |
| SELP | 0.825853 | 3.845392 | 5.97173 | 4.31E-09 | 2.00E-08 | 10.05569 |
| DNAJC3 | 0.823637 | 3.972002 | 7.457156 | 3.65E-13 | 2.97E-12 | 19.19058 |
| VCAN | 0.818539 | 4.524356 | 6.111516 | 1.92E-09 | 9.35E-09 | 10.8417 |
| ABCA13 | 0.816939 | 2.926197 | 3.791523 | 0.000167 | 0.000388 | -0.0579 |
| DHRS13 | 0.816827 | 3.941507 | 6.447856 | 2.56E-10 | 1.40E-09 | 12.79714 |
| LDHA | 0.815559 | 4.872307 | 6.812857 | 2.61E-11 | 1.61E-10 | 15.01985 |
| FLVCR2 | 0.815371 | 3.12761 | 8.139772 | 2.86E-15 | 3.20E-14 | 23.93842 |
| CAPG | 0.813179 | 4.141323 | 6.68344 | 5.94E-11 | 3.51E-10 | 14.21993 |
| FOS | 0.810061 | 3.729031 | 6.333194 | 5.14E-10 | 2.70E-09 | 12.1204 |
| MGST1 | 0.810032 | 4.471546 | 5.363988 | 1.22E-07 | 4.50E-07 | 6.825089 |
| MYL9 | 0.809206 | 3.370238 | 4.769959 | 2.39E-06 | 7.41E-06 | 3.969056 |
| SIRPB1 | 0.807841 | 3.781507 | 7.684645 | 7.51E-14 | 6.70E-13 | 20.73615 |
| TLR1 | 0.803261 | 4.2312 | 7.406995 | 5.14E-13 | 4.09E-12 | 18.85483 |
| ASGR2 | 0.80121 | 3.831224 | 7.073181 | 4.83E-12 | 3.28E-11 | 16.66762 |
| LHFPL2 | 0.798588 | 3.666454 | 8.24088 | 1.36E-15 | 1.59E-14 | 24.66929 |
| PFKFB4 | 0.79833 | 3.337984 | 9.017852 | 3.53E-18 | 6.49E-17 | 30.51262 |
| CYYR1 | 0.796785 | 3.048842 | 5.693432 | 2.07E-08 | 8.55E-08 | 8.538311 |
| WBP2 | 0.795153 | 4.185424 | 7.222887 | 1.79E-12 | 1.30E-11 | 17.63831 |
| NPL | 0.793903 | 4.292674 | 8.238853 | 1.38E-15 | 1.61E-14 | 24.65457 |
| ANKS1A | 0.78994 | 3.458101 | 7.243982 | 1.55E-12 | 1.14E-11 | 17.77643 |
| MICAL1 | 0.789796 | 3.258539 | 6.511638 | 1.73E-10 | 9.63E-10 | 13.17807 |
| SLC11A1 | 0.78577 | 4.010269 | 7.69871 | 6.81E-14 | 6.11E-13 | 20.83293 |
| ABHD5 | 0.785559 | 3.723334 | 7.41982 | 4.71E-13 | 3.77E-12 | 18.9405 |
| IFIT3 | 0.782354 | 3.978513 | 2.732475 | 0.006496 | 0.011604 | -3.42669 |
| PPP1R3B | 0.781594 | 3.549051 | 8.199234 | 1.85E-15 | 2.13E-14 | 24.3674 |
| TYROBP | 0.781306 | 5.217362 | 6.557293 | 1.31E-10 | 7.37E-10 | 13.45269 |
| EXOC6 | 0.779088 | 3.686848 | 7.991185 | 8.44E-15 | 8.82E-14 | 22.87707 |
| MERTK | 0.778438 | 2.907648 | 6.729588 | 4.44E-11 | 2.65E-10 | 14.50369 |
| TTK | 0.777486 | 3.159547 | 3.867875 | 0.000123 | 0.000293 | 0.225253 |
| CSGALNACT2 | 0.777485 | 3.554586 | 6.250529 | 8.43E-10 | 4.31E-09 | 11.63897 |
| KIF11 | 0.77645 | 3.331125 | 3.658487 | 0.000279 | 0.000629 | -0.53849 |
| TMEM45A | 0.772271 | 3.450072 | 3.864188 | 0.000125 | 0.000297 | 0.211457 |
| ADCY3 | 0.771823 | 3.616261 | 5.365944 | 1.21E-07 | 4.46E-07 | 6.834993 |
| NCF2 | 0.769034 | 4.107492 | 6.254151 | 8.25E-10 | 4.22E-09 | 11.65995 |
| LRP10 | 0.76878 | 3.552958 | 9.004997 | 3.90E-18 | 7.10E-17 | 30.41277 |
| SLC2A1 | 0.767812 | 3.630742 | 4.024395 | 6.55E-05 | 0.000162 | 0.82236 |
| VIM | 0.767514 | 5.200718 | 5.288987 | 1.80E-07 | 6.51E-07 | 6.447814 |
| BRI3 | 0.767226 | 3.959675 | 7.360057 | 7.08E-13 | 5.48E-12 | 18.54231 |
| RTN3 | 0.765785 | 3.836017 | 8.485653 | 2.17E-16 | 2.86E-15 | 26.4673 |
| PHTF1 | 0.76504 | 3.261808 | 8.356841 | 5.72E-16 | 7.01E-15 | 25.51608 |
| MBOAT2 | 0.764239 | 3.881109 | 10.28468 | 9.73E-23 | 3.92E-21 | 40.84246 |
| HIST1H2BC | 0.763621 | 3.122905 | 6.125144 | 1.77E-09 | 8.70E-09 | 10.91917 |
| ATP6V0D1 | 0.760994 | 4.287459 | 7.433799 | 4.28E-13 | 3.45E-12 | 19.03402 |
| EIF4G3 | 0.760494 | 3.409851 | 8.523914 | 1.62E-16 | 2.16E-15 | 26.75197 |
| HERC5 | 0.759943 | 4.079589 | 2.579174 | 0.010173 | 0.017495 | -3.8266 |
| XPO6 | 0.757989 | 4.219661 | 7.889476 | 1.76E-14 | 1.74E-13 | 22.15939 |
| NTNG2 | 0.757505 | 3.548908 | 8.429377 | 3.32E-16 | 4.23E-15 | 26.05036 |
| LGALS1 | 0.757349 | 4.617824 | 6.033605 | 3.02E-09 | 1.43E-08 | 10.40166 |
| EMILIN2 | 0.757191 | 4.644568 | 5.441885 | 8.08E-08 | 3.07E-07 | 7.221964 |
| TOP2A | 0.756972 | 3.440483 | 4.190721 | 3.26E-05 | 8.49E-05 | 1.481246 |
| TGFA | 0.756551 | 3.520605 | 8.232895 | 1.44E-15 | 1.68E-14 | 24.61132 |
| ENTPD1 | 0.755528 | 3.633712 | 8.05008 | 5.50E-15 | 5.90E-14 | 23.29593 |
| VAMP3 | 0.755481 | 3.718814 | 6.600479 | 9.99E-11 | 5.74E-10 | 13.71396 |
| TRIM25 | 0.753555 | 3.289482 | 8.511666 | 1.78E-16 | 2.36E-15 | 26.66073 |
| BATF | 0.753355 | 3.504373 | 8.622548 | 7.65E-17 | 1.08E-15 | 27.49027 |
| MLKL | 0.749435 | 3.800726 | 6.93194 | 1.21E-11 | 7.81E-11 | 15.76723 |
| EBI3 | 0.746747 | 2.747239 | 3.792075 | 0.000167 | 0.000388 | -0.05588 |
| TNFSF13B | 0.745725 | 4.417482 | 6.413874 | 3.15E-10 | 1.70E-09 | 12.59549 |
| WFDC1 | 0.7441 | 2.822303 | 4.539505 | 6.99E-06 | 2.03E-05 | 2.943606 |
| IGF2R | 0.743355 | 3.951693 | 6.416612 | 3.10E-10 | 1.67E-09 | 12.6117 |
| DNASE1L1 | 0.743215 | 3.879441 | 7.955212 | 1.09E-14 | 1.12E-13 | 22.62241 |
| RAB24 | 0.742781 | 4.065381 | 6.351692 | 4.60E-10 | 2.43E-09 | 12.22887 |
| RAP1GAP | 0.737408 | 3.591037 | 4.391396 | 1.36E-05 | 3.77E-05 | 2.309357 |
| SBNO2 | 0.736653 | 3.877453 | 9.734603 | 1.04E-20 | 2.83E-19 | 36.24111 |
| AGTPBP1 | 0.735589 | 3.508736 | 7.539474 | 2.07E-13 | 1.73E-12 | 19.74555 |
| LILRB2 | 0.735427 | 4.39919 | 7.352829 | 7.44E-13 | 5.72E-12 | 18.49434 |
| RHOG | 0.731778 | 4.83251 | 7.173017 | 2.49E-12 | 1.76E-11 | 17.3131 |
| PIK3CB | 0.731177 | 3.487108 | 7.0394 | 6.03E-12 | 4.02E-11 | 16.4509 |
| CFLAR | 0.730967 | 4.236254 | 7.892703 | 1.72E-14 | 1.70E-13 | 22.18205 |
| ITGA2B | 0.73082 | 2.99148 | 5.173896 | 3.26E-07 | 1.14E-06 | 5.87818 |
| ARG2 | 0.729799 | 3.966948 | 2.836898 | 0.00473 | 0.008658 | -3.14145 |
| CD82 | 0.729271 | 3.646059 | 9.275312 | 4.51E-19 | 9.56E-18 | 32.53427 |
| NFKBIZ | 0.729048 | 3.946447 | 7.763624 | 4.30E-14 | 4.01E-13 | 21.28141 |
| SLC37A3 | 0.726364 | 3.28947 | 6.030692 | 3.07E-09 | 1.45E-08 | 10.3853 |
| IL10RB | 0.72202 | 4.24914 | 7.237897 | 1.61E-12 | 1.18E-11 | 17.73656 |
| STK3 | 0.721661 | 3.011114 | 8.523705 | 1.63E-16 | 2.16E-15 | 26.75041 |
| SLCO4C1 | 0.720218 | 3.137884 | 7.587724 | 1.48E-13 | 1.26E-12 | 20.07311 |
| CAMKK2 | 0.719762 | 3.182885 | 8.407682 | 3.91E-16 | 4.86E-15 | 25.89019 |
| HMOX1 | 0.717323 | 3.641515 | 5.070399 | 5.50E-07 | 1.87E-06 | 5.375595 |
| GNB2 | 0.71489 | 3.632483 | 7.682501 | 7.63E-14 | 6.77E-13 | 20.72141 |
| JAK2 | 0.713529 | 3.756813 | 7.636314 | 1.05E-13 | 9.20E-13 | 20.40468 |
| DNTTIP1 | 0.711357 | 3.854924 | 7.401372 | 5.34E-13 | 4.24E-12 | 18.81731 |
| MTMR3 | 0.711065 | 3.878296 | 10.27151 | 1.09E-22 | 4.32E-21 | 40.73029 |
| IQGAP1 | 0.709182 | 4.543907 | 7.16885 | 2.56E-12 | 1.81E-11 | 17.28601 |
| ATXN1 | 0.708308 | 3.46609 | 8.555745 | 1.27E-16 | 1.73E-15 | 26.98953 |
| TMCO3 | 0.706094 | 3.245374 | 7.375854 | 6.36E-13 | 4.95E-12 | 18.64731 |
| YIPF1 | 0.705984 | 3.8552 | 8.605362 | 8.73E-17 | 1.22E-15 | 27.36117 |
| LACTB | 0.703525 | 3.791569 | 5.240046 | 2.32E-07 | 8.27E-07 | 6.204202 |
| CD93 | 0.698078 | 3.742154 | 6.857718 | 1.96E-11 | 1.23E-10 | 15.30013 |
| P2RY13 | 0.697532 | 4.469375 | 7.379493 | 6.20E-13 | 4.85E-12 | 18.67152 |
| NFKBIA | 0.696283 | 4.239054 | 8.13439 | 2.97E-15 | 3.32E-14 | 23.89971 |
| IRF7 | 0.695858 | 4.067463 | 4.09164 | 4.95E-05 | 0.000125 | 1.085732 |
| HIF1A | 0.695722 | 2.936614 | 4.558719 | 6.40E-06 | 1.87E-05 | 3.027315 |
| IFI6 | 0.693155 | 4.040889 | 2.296414 | 0.022043 | 0.035325 | -4.50505 |
| CSF2RA | 0.692745 | 4.145749 | 6.26709 | 7.64E-10 | 3.91E-09 | 11.73499 |
| SORL1 | 0.691923 | 4.343036 | 7.311229 | 9.86E-13 | 7.46E-12 | 18.21893 |
| OSM | 0.68921 | 3.130941 | 10.0438 | 7.69E-22 | 2.49E-20 | 38.80638 |
| BLOC1S1 | 0.688329 | 4.016928 | 6.622345 | 8.71E-11 | 5.05E-10 | 13.8468 |
| LILRA2 | 0.68572 | 3.756473 | 5.507356 | 5.69E-08 | 2.22E-07 | 7.559484 |
| SMARCD3 | 0.685173 | 3.696603 | 5.92322 | 5.69E-09 | 2.60E-08 | 9.78663 |
| CDKN2D | 0.684212 | 4.142387 | 7.626605 | 1.13E-13 | 9.81E-13 | 20.33829 |
| REPS2 | 0.683938 | 3.520647 | 8.13676 | 2.92E-15 | 3.27E-14 | 23.91675 |
| TRIB1 | 0.683626 | 3.465646 | 9.775377 | 7.41E-21 | 2.03E-19 | 36.57624 |
| ARHGAP26 | 0.683542 | 3.316621 | 7.63789 | 1.04E-13 | 9.11E-13 | 20.41546 |
| FOSL2 | 0.68256 | 3.503737 | 8.432368 | 3.24E-16 | 4.15E-15 | 26.07247 |
| SKAP2 | 0.6812 | 3.626299 | 8.540336 | 1.43E-16 | 1.93E-15 | 26.87445 |
| SLC28A3 | 0.680484 | 3.118888 | 3.891996 | 0.000112 | 0.000268 | 0.315815 |
| IFNGR1 | 0.680288 | 4.628357 | 7.968989 | 9.91E-15 | 1.02E-13 | 22.71983 |
| SLC16A6 | 0.679818 | 3.538704 | 5.929532 | 5.49E-09 | 2.51E-08 | 9.821534 |
| PLAUR | 0.678001 | 3.666056 | 6.927521 | 1.25E-11 | 8.02E-11 | 15.7393 |
| PHC2 | 0.675685 | 3.600609 | 8.532534 | 1.52E-16 | 2.04E-15 | 26.81624 |
| IFI30 | 0.671914 | 4.652322 | 6.694638 | 5.53E-11 | 3.28E-10 | 14.28863 |
| TIFA | 0.671235 | 3.337334 | 5.221168 | 2.56E-07 | 9.04E-07 | 6.110783 |
| CAMP | 0.670077 | 4.985984 | 2.401744 | 0.016661 | 0.027336 | -4.26132 |
| STAT3 | 0.669387 | 3.712091 | 8.987292 | 4.49E-18 | 8.03E-17 | 30.27541 |
| CEACAM3 | 0.667235 | 3.467905 | 9.116935 | 1.61E-18 | 3.11E-17 | 31.28577 |
| GBP5 | 0.666534 | 3.366901 | 3.080117 | 0.002177 | 0.004234 | -2.43694 |
| IFI35 | 0.666353 | 4.040142 | 3.705089 | 0.000234 | 0.000532 | -0.372 |
| ANXA1 | 0.665585 | 4.772449 | 5.291195 | 1.78E-07 | 6.44E-07 | 6.458855 |
| DUSP1 | 0.663406 | 3.342127 | 8.173689 | 2.23E-15 | 2.53E-14 | 24.18282 |
| FKBP1A | 0.661675 | 4.224066 | 7.345156 | 7.83E-13 | 6.01E-12 | 18.44344 |
| RBPJ | 0.661564 | 3.432426 | 6.740108 | 4.15E-11 | 2.50E-10 | 14.56861 |
| HIST1H2BK | 0.660641 | 3.821899 | 7.209478 | 1.95E-12 | 1.41E-11 | 17.55069 |
| PDZD8 | 0.659862 | 3.338427 | 7.165393 | 2.62E-12 | 1.84E-11 | 17.26354 |
| FECH | 0.658629 | 4.258234 | 3.409925 | 0.000699 | 0.001484 | -1.3926 |
| MARCO | 0.657593 | 3.158929 | 6.345022 | 4.78E-10 | 2.52E-09 | 12.18972 |
| TSEN34 | 0.656842 | 3.895758 | 7.365622 | 6.82E-13 | 5.28E-12 | 18.57928 |
| MVP | 0.656083 | 3.569738 | 6.788446 | 3.05E-11 | 1.87E-10 | 14.86798 |
| JDP2 | 0.655312 | 3.672344 | 7.108255 | 3.83E-12 | 2.63E-11 | 16.89354 |
| TLE3 | 0.653548 | 3.421046 | 7.919436 | 1.42E-14 | 1.42E-13 | 22.37005 |
| PTAFR | 0.6528 | 3.665066 | 7.270557 | 1.30E-12 | 9.63E-12 | 17.9509 |
| ACOX1 | 0.652509 | 3.261609 | 8.473832 | 2.37E-16 | 3.10E-15 | 26.37954 |
| PGK1 | 0.651766 | 4.152052 | 6.338104 | 4.99E-10 | 2.62E-09 | 12.14916 |
| MAFG | 0.650755 | 3.393858 | 9.795042 | 6.28E-21 | 1.74E-19 | 36.73822 |
| FURIN | 0.65063 | 4.066787 | 7.315062 | 9.60E-13 | 7.28E-12 | 18.24425 |
| KL | 0.650624 | 2.957405 | 5.2313 | 2.43E-07 | 8.62E-07 | 6.160885 |
| TBC1D14 | 0.65027 | 3.788002 | 6.161517 | 1.43E-09 | 7.10E-09 | 11.12668 |
| LYST | 0.648229 | 3.419583 | 6.360203 | 4.37E-10 | 2.32E-09 | 12.27887 |
| ELL2 | 0.647354 | 3.436667 | 7.817907 | 2.93E-14 | 2.80E-13 | 21.65873 |
| PLEKHO2 | 0.646551 | 4.14754 | 8.599368 | 9.13E-17 | 1.27E-15 | 27.31619 |
| C1RL | 0.644155 | 3.473849 | 9.891196 | 2.80E-21 | 8.40E-20 | 37.53344 |
| CTSB | 0.643709 | 4.39147 | 6.503088 | 1.83E-10 | 1.01E-09 | 13.12681 |
| HMBS | 0.642656 | 3.727539 | 5.156104 | 3.57E-07 | 1.24E-06 | 5.791128 |
| PLXDC2 | 0.642564 | 3.65813 | 6.88544 | 1.64E-11 | 1.04E-10 | 15.4741 |
| DUSP3 | 0.642373 | 3.417247 | 7.974784 | 9.50E-15 | 9.89E-14 | 22.76085 |
| SCO2 | 0.642184 | 4.062706 | 4.054214 | 5.79E-05 | 0.000145 | 0.938641 |
| IRS2 | 0.640998 | 3.155668 | 7.21087 | 1.94E-12 | 1.40E-11 | 17.55977 |
| TRPM6 | 0.640025 | 2.806863 | 8.44743 | 2.90E-16 | 3.73E-15 | 26.18388 |
| QSOX1 | 0.639499 | 3.294585 | 9.556691 | 4.55E-20 | 1.10E-18 | 34.79024 |
| LAPTM4B | 0.638814 | 3.200994 | 6.747302 | 3.97E-11 | 2.39E-10 | 14.61305 |
| ANLN | 0.638322 | 2.965781 | 4.406415 | 1.27E-05 | 3.55E-05 | 2.372781 |
| MSRA | 0.637467 | 3.586224 | 7.236142 | 1.63E-12 | 1.19E-11 | 17.72506 |
| RHOT1 | 0.637117 | 3.697698 | 6.763688 | 3.58E-11 | 2.17E-10 | 14.71442 |
| SLC9A8 | 0.635713 | 3.83183 | 8.384869 | 4.64E-16 | 5.71E-15 | 25.72211 |
| VASP | 0.635146 | 4.168381 | 8.058852 | 5.16E-15 | 5.57E-14 | 23.35852 |
| DTL | 0.633696 | 3.489077 | 3.248983 | 0.001232 | 0.002495 | -1.91497 |
| GNG5 | 0.633205 | 4.130867 | 8.579788 | 1.06E-16 | 1.45E-15 | 27.16941 |
| ACPP | 0.632993 | 3.228487 | 7.391462 | 5.72E-13 | 4.51E-12 | 18.75123 |
| USP32 | 0.629414 | 3.095711 | 9.176962 | 9.94E-19 | 1.98E-17 | 31.75714 |
| CEBPE | 0.629028 | 3.505073 | 4.936043 | 1.07E-06 | 3.51E-06 | 4.736918 |
| CCL20 | 0.628971 | 2.57877 | 2.975583 | 0.003058 | 0.005789 | -2.7466 |
| IFITM2 | 0.628845 | 5.337189 | 9.201738 | 8.15E-19 | 1.64E-17 | 31.95235 |
| ERMAP | 0.626555 | 4.092821 | 5.560141 | 4.28E-08 | 1.69E-07 | 7.834215 |
| ADAM8 | 0.626034 | 4.182564 | 6.825582 | 2.41E-11 | 1.49E-10 | 15.0992 |
| ABTB1 | 0.625407 | 3.349867 | 6.284054 | 6.90E-10 | 3.55E-09 | 11.83356 |
| UBE2H | 0.625095 | 3.909391 | 7.532273 | 2.17E-13 | 1.81E-12 | 19.6968 |
| RP2 | 0.625092 | 3.488004 | 5.904961 | 6.32E-09 | 2.85E-08 | 9.685853 |
| GPR27 | 0.624263 | 3.348483 | 7.772031 | 4.06E-14 | 3.80E-13 | 21.33971 |
| TNFRSF10C | 0.624162 | 3.450022 | 7.661053 | 8.87E-14 | 7.79E-13 | 20.57414 |
| SNX3 | 0.623452 | 4.370509 | 7.78676 | 3.65E-14 | 3.45E-13 | 21.44197 |
| WARS | 0.623339 | 3.540159 | 3.521546 | 0.000466 | 0.001014 | -1.01614 |
| LPGAT1 | 0.622488 | 3.468426 | 6.501124 | 1.85E-10 | 1.02E-09 | 13.11505 |
| ITGAX | 0.621997 | 3.680175 | 7.577628 | 1.59E-13 | 1.34E-12 | 20.00443 |
| DUSP13 | 0.620326 | 2.941642 | 8.060907 | 5.09E-15 | 5.51E-14 | 23.37319 |
| SRGN | 0.619416 | 5.139461 | 6.069562 | 2.45E-09 | 1.18E-08 | 10.60413 |
| PLD1 | 0.618089 | 2.961547 | 7.740637 | 5.06E-14 | 4.65E-13 | 21.12225 |
| CYBRD1 | 0.617522 | 3.580191 | 5.459231 | 7.37E-08 | 2.82E-07 | 7.311034 |
| TNFRSF1A | 0.616954 | 3.822705 | 6.317672 | 5.64E-10 | 2.94E-09 | 12.02959 |
| TM6SF1 | 0.616427 | 3.857869 | 5.608579 | 3.29E-08 | 1.32E-07 | 8.088374 |
| TMEM56 | 0.615858 | 3.511839 | 4.958981 | 9.56E-07 | 3.16E-06 | 4.844851 |
| OLR1 | 0.615265 | 3.014121 | 3.493098 | 0.000517 | 0.001119 | -1.11318 |
| SLC14A1 | 0.614785 | 4.128474 | 2.351016 | 0.019089 | 0.03102 | -4.38004 |
| IGF1R | 0.611563 | 3.656243 | 8.414059 | 3.72E-16 | 4.66E-15 | 25.93724 |
| S100A6 | 0.610475 | 4.658922 | 6.037955 | 2.94E-09 | 1.40E-08 | 10.4261 |
| CDKN3 | 0.610463 | 3.315926 | 3.760647 | 0.000188 | 0.000435 | -0.17089 |
| PNPLA6 | 0.610225 | 3.354683 | 6.790897 | 3.01E-11 | 1.84E-10 | 14.88321 |
| HIST1H2BG | 0.608742 | 3.686945 | 4.189867 | 3.27E-05 | 8.52E-05 | 1.477799 |
| MMP25 | 0.608663 | 3.979639 | 8.623863 | 7.57E-17 | 1.08E-15 | 27.50016 |
| HIST1H2BE | 0.608569 | 3.308036 | 6.00496 | 3.56E-09 | 1.67E-08 | 10.24111 |
| GALNT4 | 0.607332 | 3.039469 | 6.127883 | 1.74E-09 | 8.57E-09 | 10.93476 |
| TOR1B | 0.606409 | 3.310359 | 5.416316 | 9.25E-08 | 3.48E-07 | 7.091129 |
| EXT1 | 0.606404 | 3.102337 | 7.065364 | 5.08E-12 | 3.44E-11 | 16.61739 |
| DHCR7 | 0.605607 | 3.15377 | 7.356234 | 7.27E-13 | 5.60E-12 | 18.51693 |
| MXD3 | 0.605219 | 3.085515 | 8.842918 | 1.39E-17 | 2.29E-16 | 29.16276 |
| GRAMD1A | 0.605111 | 3.629751 | 5.723827 | 1.75E-08 | 7.32E-08 | 8.700937 |
| ARID3A | 0.6051 | 3.822304 | 8.61545 | 8.08E-17 | 1.14E-15 | 27.43693 |
| TPST2 | 0.604968 | 4.481247 | 8.863242 | 1.19E-17 | 1.99E-16 | 29.31859 |
| CTNNA1 | 0.604959 | 3.642403 | 9.051043 | 2.71E-18 | 5.05E-17 | 30.77093 |
| MSRB3 | 0.604487 | 3.01204 | 7.597445 | 1.38E-13 | 1.18E-12 | 20.13931 |
| PBX1 | 0.602791 | 3.267255 | 4.491692 | 8.68E-06 | 2.49E-05 | 2.736726 |
| CEBPA | 0.602538 | 4.040174 | 5.529568 | 5.05E-08 | 1.98E-07 | 7.674807 |
| IGF2BP3 | 0.599775 | 4.023352 | 4.301562 | 2.02E-05 | 5.47E-05 | 1.934181 |
| IL13RA1 | 0.599773 | 3.642454 | 5.134662 | 3.98E-07 | 1.38E-06 | 5.686577 |
| KIF14 | 0.599465 | 3.37811 | 5.046378 | 6.20E-07 | 2.10E-06 | 5.260265 |
| FAS | 0.598432 | 3.378707 | 4.51424 | 7.84E-06 | 2.27E-05 | 2.834038 |
| SCPEP1 | 0.59731 | 3.524581 | 6.204517 | 1.11E-09 | 5.57E-09 | 11.37337 |
| PGM2 | 0.595656 | 3.463785 | 5.286481 | 1.83E-07 | 6.59E-07 | 6.435294 |
| LDLR | 0.594337 | 3.439415 | 7.811384 | 3.07E-14 | 2.93E-13 | 21.61328 |
| TALDO1 | 0.593741 | 3.844255 | 7.556875 | 1.83E-13 | 1.54E-12 | 19.86349 |
| ZFP36 | 0.593062 | 4.383708 | 6.679781 | 6.08E-11 | 3.59E-10 | 14.1975 |
| MAP3K5 | 0.59178 | 3.401872 | 7.404718 | 5.22E-13 | 4.15E-12 | 18.83964 |
| DMXL2 | 0.591501 | 3.219815 | 5.593881 | 3.57E-08 | 1.43E-07 | 8.011046 |
| CREG1 | 0.589595 | 4.044814 | 5.785478 | 1.24E-08 | 5.34E-08 | 9.033135 |
| KIF4A | 0.58951 | 2.780618 | 5.414482 | 9.34E-08 | 3.51E-07 | 7.081765 |
| HSPA6 | 0.588807 | 3.459937 | 6.04142 | 2.88E-09 | 1.37E-08 | 10.44558 |
| CLIC1 | 0.588628 | 4.275606 | 6.620646 | 8.80E-11 | 5.09E-10 | 13.83646 |
| KRT23 | 0.587409 | 3.262024 | 4.376413 | 1.45E-05 | 4.01E-05 | 2.246279 |
| OAT | 0.587274 | 3.661499 | 5.222626 | 2.54E-07 | 8.98E-07 | 6.117985 |
| IMPDH1 | 0.587094 | 4.033204 | 7.936899 | 1.25E-14 | 1.26E-13 | 22.49312 |
| PLSCR4 | 0.586009 | 2.752647 | 5.352765 | 1.29E-07 | 4.75E-07 | 6.768331 |
| MAN2A2 | 0.58599 | 3.704398 | 7.888448 | 1.77E-14 | 1.75E-13 | 22.15218 |
| NCF1 | 0.58578 | 4.297403 | 5.139185 | 3.89E-07 | 1.35E-06 | 5.708599 |
| TRIAP1 | -0.69677 | 2.836901 | -9.61842 | 2.73E-20 | 6.92E-19 | 35.29156 |
| TDRD3 | -0.69681 | 2.154118 | -7.82459 | 2.79E-14 | 2.68E-13 | 21.70536 |
| CD5 | -0.69853 | 2.574434 | -6.95076 | 1.07E-11 | 6.94E-11 | 15.88634 |
| CETN3 | -0.69917 | 2.264099 | -5.67768 | 2.25E-08 | 9.28E-08 | 8.454353 |
| ITGA4 | -0.69954 | 2.872075 | -10.3643 | 4.88E-23 | 2.09E-21 | 41.52225 |
| ZNF32 | -0.69963 | 2.861428 | -9.53368 | 5.50E-20 | 1.32E-18 | 34.60395 |
| GZMH | -0.70282 | 3.110746 | -5.61078 | 3.25E-08 | 1.31E-07 | 8.099985 |
| TMEM42 | -0.70316 | 2.910675 | -11.9385 | 2.96E-29 | 3.35E-27 | 55.63721 |
| ARL5A | -0.70334 | 2.682482 | -9.58725 | 3.54E-20 | 8.85E-19 | 35.03811 |
| PTPRCAP | -0.70449 | 3.263616 | -7.61314 | 1.24E-13 | 1.07E-12 | 20.24635 |
| RBL2 | -0.70514 | 3.517444 | -9.58633 | 3.56E-20 | 8.87E-19 | 35.03063 |
| RPS15A | -0.70523 | 4.467753 | -6.82736 | 2.38E-11 | 1.47E-10 | 15.1103 |
| CAMK4 | -0.70563 | 2.49348 | -12.0808 | 7.67E-30 | 1.06E-27 | 56.97079 |
| FHIT | -0.70589 | 3.083068 | -7.35997 | 7.08E-13 | 5.48E-12 | 18.54174 |
| CASD1 | -0.70691 | 2.705726 | -10.5863 | 6.99E-24 | 3.43E-22 | 43.43683 |
| TXNIP | -0.70742 | 3.931387 | -3.55865 | 0.000406 | 0.000892 | -0.88843 |
| TNFRSF25 | -0.70744 | 3.031539 | -10.4329 | 2.68E-23 | 1.20E-21 | 42.11155 |
| RPUSD2 | -0.70752 | 2.904705 | -13.6141 | 2.14E-36 | 8.37E-34 | 71.87485 |
| CCR3 | -0.7078 | 2.961058 | -8.69761 | 4.30E-17 | 6.41E-16 | 28.05638 |
| PHF14 | -0.70845 | 2.563208 | -9.4623 | 9.87E-20 | 2.28E-18 | 34.02809 |
| ZBTB5 | -0.71092 | 2.179428 | -8.05698 | 5.24E-15 | 5.63E-14 | 23.34518 |
| DDHD2 | -0.71299 | 3.804667 | -9.19974 | 8.28E-19 | 1.66E-17 | 31.93659 |
| VPREB3 | -0.71604 | 3.026478 | -7.4491 | 3.85E-13 | 3.13E-12 | 19.13656 |
| PPIL3 | -0.71627 | 3.119998 | -8.13052 | 3.06E-15 | 3.41E-14 | 23.87192 |
| BIRC3 | -0.71667 | 2.986347 | -10.2916 | 9.16E-23 | 3.71E-21 | 40.90145 |
| TCL1A | -0.72157 | 3.029967 | -8.01242 | 7.24E-15 | 7.64E-14 | 23.02783 |
| SIGIRR | -0.72199 | 2.807707 | -8.93725 | 6.66E-18 | 1.15E-16 | 29.88822 |
| RFTN1 | -0.72203 | 2.947742 | -13.0859 | 4.30E-34 | 1.35E-31 | 66.63688 |
| AP1S2 | -0.72353 | 3.509681 | -8.40805 | 3.90E-16 | 4.86E-15 | 25.89294 |
| NOL11 | -0.72377 | 3.263111 | -8.79438 | 2.03E-17 | 3.19E-16 | 28.79167 |
| PDCL3 | -0.7257 | 3.002744 | -8.43686 | 3.14E-16 | 4.03E-15 | 26.10565 |
| UFSP2 | -0.72812 | 2.45313 | -6.98513 | 8.59E-12 | 5.60E-11 | 16.10456 |
| GNPDA2 | -0.72855 | 2.20136 | -11.5064 | 1.70E-27 | 1.55E-25 | 51.64186 |
| ZNF302 | -0.72856 | 2.601764 | -11.1507 | 4.46E-26 | 3.10E-24 | 48.41952 |
| TRIB2 | -0.73314 | 3.311528 | -11.9751 | 2.10E-29 | 2.56E-27 | 55.97911 |
| LAT | -0.73385 | 3.168405 | -12.959 | 1.51E-33 | 4.30E-31 | 65.39431 |
| SOX4 | -0.73421 | 3.156824 | -8.68725 | 4.65E-17 | 6.88E-16 | 27.97808 |
| EIF4B | -0.73497 | 3.51132 | -7.44454 | 3.98E-13 | 3.22E-12 | 19.10596 |
| TMEM203 | -0.73563 | 3.437053 | -11.3113 | 1.03E-26 | 7.97E-25 | 49.86737 |
| GZMA | -0.73613 | 3.640419 | -7.60455 | 1.32E-13 | 1.13E-12 | 20.18777 |
| CD28 | -0.73646 | 2.783144 | -8.3673 | 5.29E-16 | 6.50E-15 | 25.59287 |
| PMPCB | -0.73652 | 2.713246 | -5.78139 | 1.27E-08 | 5.45E-08 | 9.010987 |
| MRFAP1L1 | -0.73831 | 3.032298 | -12.0243 | 1.31E-29 | 1.71E-27 | 56.44046 |
| CCL5 | -0.73838 | 3.358984 | -8.42677 | 3.38E-16 | 4.29E-15 | 26.0311 |
| SERINC5 | -0.73952 | 2.079201 | -7.61419 | 1.23E-13 | 1.06E-12 | 20.25352 |
| TCF7 | -0.74074 | 3.479475 | -9.14461 | 1.29E-18 | 2.54E-17 | 31.50283 |
| TNFRSF17 | -0.74934 | 2.723493 | -5.63594 | 2.84E-08 | 1.15E-07 | 8.232808 |
| RSL1D1 | -0.74946 | 3.428927 | -8.83531 | 1.48E-17 | 2.41E-16 | 29.10447 |
| GATA3 | -0.75103 | 2.803545 | -10.7165 | 2.21E-24 | 1.17E-22 | 44.57202 |
| UBASH3A | -0.75175 | 2.325493 | -11.8239 | 8.75E-29 | 9.23E-27 | 54.56946 |
| IMP3 | -0.75789 | 3.309083 | -11.1966 | 2.94E-26 | 2.10E-24 | 48.832 |
| RPL27 | -0.76032 | 4.045527 | -7.27097 | 1.29E-12 | 9.61E-12 | 17.95364 |
| SIRPG | -0.76387 | 3.361749 | -11.8127 | 9.72E-29 | 1.01E-26 | 54.46545 |
| ABCE1 | -0.76616 | 3.985039 | -7.37878 | 6.23E-13 | 4.86E-12 | 18.6668 |
| MCUB | -0.7677 | 2.943154 | -11.4682 | 2.42E-27 | 2.12E-25 | 51.29353 |
| UPF3A | -0.76827 | 2.287892 | -9.70569 | 1.32E-20 | 3.49E-19 | 36.00406 |
| SRP72 | -0.76889 | 3.313147 | -10.1324 | 3.61E-22 | 1.28E-20 | 39.55176 |
| ZNF260 | -0.77084 | 2.868576 | -13.9986 | 4.24E-38 | 2.49E-35 | 75.74917 |
| MPHOSPH10 | -0.77146 | 2.834912 | -9.80281 | 5.89E-21 | 1.65E-19 | 36.80224 |
| NAP1L1 | -0.7723 | 2.918378 | -11.2886 | 1.27E-26 | 9.66E-25 | 49.66189 |
| GIMAP1 | -0.77632 | 3.287596 | -12.5647 | 7.25E-32 | 1.31E-29 | 61.57256 |
| SLC38A1 | -0.77656 | 3.107622 | -10.3618 | 4.99E-23 | 2.11E-21 | 41.50071 |
| ETS1 | -0.77658 | 3.351399 | -11.2945 | 1.20E-26 | 9.22E-25 | 49.71521 |
| MAGEH1 | -0.77775 | 2.678507 | -11.3448 | 7.56E-27 | 6.12E-25 | 50.17018 |
| ZNF121 | -0.77909 | 3.576717 | -6.72348 | 4.61E-11 | 2.75E-10 | 14.46602 |
| UBE2Q2 | -0.78033 | 2.299943 | -14.1035 | 1.44E-38 | 9.67E-36 | 76.81453 |
| KLRF1 | -0.78401 | 2.805725 | -12.9465 | 1.71E-33 | 4.72E-31 | 65.27269 |
| GLOD4 | -0.78425 | 2.907451 | -9.71517 | 1.22E-20 | 3.27E-19 | 36.08174 |
| USP44 | -0.78829 | 2.091073 | -7.09954 | 4.06E-12 | 2.78E-11 | 16.83729 |
| CHI3L2 | -0.78996 | 3.152303 | -10.189 | 2.22E-22 | 8.37E-21 | 40.02972 |
| CTSW | -0.79332 | 2.602342 | -9.57934 | 3.77E-20 | 9.33E-19 | 34.97387 |
| MYOM2 | -0.7943 | 2.595033 | -8.0112 | 7.30E-15 | 7.68E-14 | 23.01912 |
| RPS25 | -0.79511 | 4.568158 | -9.31679 | 3.23E-19 | 6.98E-18 | 32.86382 |
| CLIC3 | -0.79546 | 2.440305 | -7.70928 | 6.32E-14 | 5.70E-13 | 20.90576 |
| ZNF395 | -0.79901 | 2.641265 | -6.3284 | 5.29E-10 | 2.77E-09 | 12.09232 |
| SNRPA1 | -0.80021 | 3.120763 | -5.84636 | 8.80E-09 | 3.88E-08 | 9.364289 |
| TCEAL8 | -0.80255 | 2.927832 | -9.90857 | 2.42E-21 | 7.33E-20 | 37.67768 |
| MATK | -0.80307 | 2.504379 | -14.1647 | 7.68E-39 | 5.55E-36 | 77.43762 |
| HLA-DPA1 | -0.80315 | 3.741066 | -12.0683 | 8.64E-30 | 1.18E-27 | 56.85334 |
| USPL1 | -0.8043 | 2.758299 | -6.7631 | 3.59E-11 | 2.17E-10 | 14.71075 |
| BACH2 | -0.80518 | 2.408762 | -11.27 | 1.50E-26 | 1.14E-24 | 49.49412 |
| TRAC | -0.80607 | 2.991048 | -13.2846 | 5.92E-35 | 1.99E-32 | 68.59487 |
| STAT4 | -0.8061 | 2.416702 | -12.7097 | 1.76E-32 | 3.75E-30 | 62.97144 |
| ZNF266 | -0.80634 | 2.612459 | -9.23494 | 6.24E-19 | 1.29E-17 | 32.21453 |
| KLRG1 | -0.80665 | 2.558514 | -13.7839 | 3.81E-37 | 1.70E-34 | 73.57951 |
| TRMT61B | -0.80836 | 3.810796 | -8.86811 | 1.14E-17 | 1.92E-16 | 29.35596 |
| PTPN4 | -0.80938 | 2.597146 | -12.2802 | 1.14E-30 | 1.75E-28 | 58.85491 |
| RPL13A | -0.81087 | 3.514259 | -6.89131 | 1.58E-11 | 1.00E-10 | 15.51099 |
| MTA1 | -0.8119 | 2.969448 | -6.00119 | 3.64E-09 | 1.70E-08 | 10.22004 |
| NLRC3 | -0.81211 | 2.630214 | -14.3421 | 1.22E-39 | 1.05E-36 | 79.25165 |
| ID3 | -0.81474 | 2.873957 | -14.0266 | 3.18E-38 | 1.99E-35 | 76.03348 |
| PVRIG | -0.81479 | 3.300767 | -9.47449 | 8.93E-20 | 2.08E-18 | 34.12622 |
| FLT3LG | -0.81623 | 2.942813 | -9.66582 | 1.85E-20 | 4.79E-19 | 35.67801 |
| ANP32B | -0.81681 | 3.594598 | -4.76488 | 2.45E-06 | 7.58E-06 | 3.945958 |
| RPL7A | -0.81802 | 3.610323 | -8.64775 | 6.31E-17 | 9.14E-16 | 27.67997 |
| CD3D | -0.82786 | 3.6226 | -11.5793 | 8.64E-28 | 8.54E-26 | 52.3096 |
| EPHX2 | -0.83141 | 2.598419 | -12.7538 | 1.14E-32 | 2.55E-30 | 63.39771 |
| MAL | -0.83389 | 3.386518 | -12.2133 | 2.16E-30 | 3.17E-28 | 58.221 |
| KLRD1 | -0.83495 | 2.698119 | -10.7181 | 2.18E-24 | 1.16E-22 | 44.58575 |
| RPL32 | -0.83776 | 3.975757 | -6.7112 | 4.99E-11 | 2.96E-10 | 14.3904 |
| BIN1 | -0.83874 | 3.383693 | -10.4824 | 1.74E-23 | 8.05E-22 | 42.53735 |
| CEBPZ | -0.83896 | 3.032733 | -10.2675 | 1.13E-22 | 4.45E-21 | 40.69611 |
| TOX | -0.839 | 3.362586 | -8.18591 | 2.04E-15 | 2.32E-14 | 24.27105 |
| CLC | -0.83905 | 3.70338 | -7.75139 | 4.69E-14 | 4.34E-13 | 21.19665 |
| FGFBP2 | -0.84143 | 2.42287 | -10.4449 | 2.42E-23 | 1.09E-21 | 42.21426 |
| PATZ1 | -0.84447 | 3.037447 | -5.81901 | 1.03E-08 | 4.48E-08 | 9.215147 |
| TRAT1 | -0.84483 | 2.555549 | -12.931 | 2.00E-33 | 5.20E-31 | 65.12097 |
| KLHL3 | -0.84557 | 2.863483 | -15.2344 | 1.04E-43 | 2.44E-40 | 88.51626 |
| TXK | -0.84581 | 2.723947 | -9.17605 | 1.00E-18 | 1.99E-17 | 31.74997 |
| KLRC3 | -0.84605 | 2.471906 | -13.6054 | 2.34E-36 | 8.78E-34 | 71.78785 |
| P2RY10 | -0.84682 | 2.362831 | -12.9379 | 1.86E-33 | 5.00E-31 | 65.18805 |
| NOC3L | -0.84698 | 2.860488 | -10.1828 | 2.34E-22 | 8.79E-21 | 39.97726 |
| RPL3 | -0.85248 | 4.25932 | -7.62962 | 1.10E-13 | 9.61E-13 | 20.35893 |
| EIF3F | -0.85333 | 3.478672 | -12.1156 | 5.51E-30 | 7.72E-28 | 57.29828 |
| OCIAD2 | -0.85539 | 3.362602 | -12.6903 | 2.13E-32 | 4.25E-30 | 62.78366 |
| RPS3 | -0.85993 | 4.516062 | -6.20435 | 1.11E-09 | 5.58E-09 | 11.37241 |
| MAN1C1 | -0.86338 | 2.745123 | -12.8656 | 3.80E-33 | 8.93E-31 | 64.48371 |
| TC2N | -0.86607 | 2.720875 | -12.0104 | 1.50E-29 | 1.90E-27 | 56.30953 |
| CHMP7 | -0.86637 | 2.828511 | -12.6222 | 4.14E-32 | 7.77E-30 | 62.12666 |
| ENOPH1 | -0.86985 | 2.374341 | -11.2107 | 2.58E-26 | 1.88E-24 | 48.95876 |
| ABLIM1 | -0.86994 | 3.449317 | -11.218 | 2.41E-26 | 1.78E-24 | 49.02454 |
| BCL11A | -0.87205 | 2.408372 | -8.66053 | 5.72E-17 | 8.33E-16 | 27.77628 |
| SKAP1 | -0.87491 | 3.402087 | -12.4744 | 1.74E-31 | 3.03E-29 | 60.70674 |
| WWP1 | -0.89126 | 2.678793 | -10.3054 | 8.13E-23 | 3.35E-21 | 41.01928 |
| RPL10A | -0.89255 | 4.445338 | -8.1176 | 3.36E-15 | 3.74E-14 | 23.77907 |
| IL2RB | -0.89323 | 3.098936 | -9.36337 | 2.21E-19 | 4.91E-18 | 33.23511 |
| GZMK | -0.89771 | 3.039541 | -9.64545 | 2.19E-20 | 5.57E-19 | 35.51174 |
| GNLY | -0.89817 | 2.942666 | -8.80361 | 1.89E-17 | 2.99E-16 | 28.8621 |
| EVL | -0.9017 | 3.275175 | -12.8512 | 4.38E-33 | 1.00E-30 | 64.34375 |
| EIF3A | -0.90829 | 2.846017 | -7.40996 | 5.04E-13 | 4.02E-12 | 18.87464 |
| DYRK2 | -0.91226 | 2.905125 | -12.9175 | 2.28E-33 | 5.79E-31 | 64.98879 |
| LCK | -0.92275 | 2.96648 | -11.0409 | 1.21E-25 | 7.71E-24 | 47.43791 |
| HLA-DRA | -0.92564 | 3.970029 | -10.0727 | 6.01E-22 | 2.01E-20 | 39.04883 |
| LEPROTL1 | -0.92611 | 3.235787 | -12.0665 | 8.80E-30 | 1.18E-27 | 56.8363 |
| TRBC1 | -0.92899 | 2.964102 | -11.4977 | 1.84E-27 | 1.65E-25 | 51.56257 |
| LEF1 | -0.9344 | 3.686757 | -11.321 | 9.40E-27 | 7.41E-25 | 49.95529 |
| SNRPN | -0.93475 | 3.345042 | -10.1288 | 3.72E-22 | 1.31E-20 | 39.52117 |
| GPR18 | -0.93503 | 2.813398 | -15.7159 | 6.04E-46 | 2.83E-42 | 93.60557 |
| GNL3 | -0.93639 | 2.217389 | -8.8448 | 1.37E-17 | 2.27E-16 | 29.17715 |
| FCER1A | -0.94614 | 2.670209 | -11.6445 | 4.70E-28 | 4.75E-26 | 52.90942 |
| LDHB | -0.94744 | 3.832889 | -10.8143 | 9.25E-25 | 5.17E-23 | 45.43029 |
| LGALS2 | -0.94744 | 2.868923 | -9.30775 | 3.47E-19 | 7.47E-18 | 32.79188 |
| GIMAP6 | -0.95023 | 2.961015 | -10.8258 | 8.35E-25 | 4.72E-23 | 45.53131 |
| PLEKHA1 | -0.95204 | 2.596786 | -14.7628 | 1.52E-41 | 2.37E-38 | 83.59096 |
| NOG | -0.95752 | 2.355226 | -13.426 | 1.43E-35 | 5.17E-33 | 69.99756 |
| GIMAP7 | -0.95935 | 3.465714 | -12.9873 | 1.14E-33 | 3.35E-31 | 65.67113 |
| NAE1 | -0.95971 | 3.30131 | -12.8898 | 3.00E-33 | 7.21E-31 | 64.71937 |
| TBC1D4 | -0.97106 | 2.617771 | -14.3951 | 7.06E-40 | 6.63E-37 | 79.79567 |
| NCR3 | -0.97275 | 2.529214 | -13.9548 | 6.65E-38 | 3.67E-35 | 75.30479 |
| LDLRAP1 | -0.97808 | 2.945728 | -11.5017 | 1.77E-27 | 1.60E-25 | 51.59928 |
| NMT2 | -0.98336 | 2.488965 | -10.7774 | 1.29E-24 | 7.10E-23 | 45.10525 |
| ITM2A | -0.98348 | 3.451808 | -12.2034 | 2.38E-30 | 3.43E-28 | 58.12773 |
| RASGRP1 | -0.988 | 3.007262 | -12.6324 | 3.75E-32 | 7.18E-30 | 62.22484 |
| ARL4C | -0.98928 | 2.549591 | -12.6074 | 4.79E-32 | 8.81E-30 | 61.98341 |
| RPS4Y1 | -0.99059 | 3.213905 | -5.07263 | 5.44E-07 | 1.85E-06 | 5.386347 |
| TMEM263 | -0.99591 | 2.844905 | -12.994 | 1.07E-33 | 3.24E-31 | 65.73628 |
| ITK | -0.99802 | 3.380955 | -13.658 | 1.37E-36 | 5.60E-34 | 72.31426 |
| RPL13 | -0.99914 | 4.152563 | -8.98266 | 4.66E-18 | 8.29E-17 | 30.23954 |
| IL7R | -1.00666 | 3.759367 | -12.6486 | 3.20E-32 | 6.26E-30 | 62.3807 |
| CD160 | -1.01961 | 2.238493 | -12.9049 | 2.58E-33 | 6.38E-31 | 64.86662 |
| ANKRD36B | -1.03808 | 2.3204 | -7.98924 | 8.56E-15 | 8.93E-14 | 22.86326 |
| NELL2 | -1.04771 | 2.628477 | -15.0724 | 5.80E-43 | 1.09E-39 | 86.81768 |
| CD2 | -1.06103 | 3.330482 | -13.8692 | 1.60E-37 | 8.32E-35 | 74.43973 |
| CD52 | -1.06706 | 4.149901 | -9.34398 | 2.59E-19 | 5.66E-18 | 33.08039 |
| HLA-DMB | -1.07171 | 3.207421 | -13.7848 | 3.78E-37 | 1.70E-34 | 73.58842 |
| RPL5 | -1.08903 | 3.529719 | -6.85048 | 2.05E-11 | 1.29E-10 | 15.25483 |
| LBH | -1.09465 | 3.250244 | -14.1982 | 5.43E-39 | 4.25E-36 | 77.77927 |
| CLEC2D | -1.10135 | 3.26543 | -11.943 | 2.84E-29 | 3.25E-27 | 55.67859 |
| CD27 | -1.10939 | 3.16003 | -11.1107 | 6.41E-26 | 4.30E-24 | 48.06137 |
| MALT1 | -1.11971 | 3.375911 | -15.2977 | 5.31E-44 | 1.66E-40 | 89.18129 |
| LOC93622 | -1.13466 | 3.548623 | -10.3548 | 5.30E-23 | 2.23E-21 | 41.44147 |
| ZAP70 | -1.203 | 2.604757 | -7.01819 | 6.92E-12 | 4.59E-11 | 16.31524 |
| E2F5 | -1.20588 | 2.553745 | -8.30536 | 8.40E-16 | 1.01E-14 | 25.13904 |
| KLRB1 | -1.21784 | 3.053399 | -15.835 | 1.67E-46 | 1.57E-42 | 94.87296 |
| LRRN3 | -1.25113 | 2.87902 | -14.7023 | 2.86E-41 | 3.36E-38 | 82.96349 |
| HLA-DQA1 | -1.30609 | 3.172274 | -11.5573 | 1.06E-27 | 1.02E-25 | 52.10819 |
| RPL15 | -1.31978 | 3.816097 | -7.83796 | 2.54E-14 | 2.45E-13 | 21.79866 |
| CD3E | -1.33064 | 2.762229 | -8.87279 | 1.10E-17 | 1.85E-16 | 29.39186 |
